# Homopolymerization and copolymerization of ε-caprolactone and l-lactide organocatalyzed by carboxylic acids with mono-, di-, and tri-functionality

**DOI:** 10.1039/d5ra07333b

**Published:** 2025-11-06

**Authors:** Juan Pablo Aldaba-Ramos, Miriam P. Barrera-Nava, José Bonilla Cruz, Alejandro Aparicio-Saguilán, Aurelio Ramírez-Hernández, José E. Báez

**Affiliations:** a Department of Chemistry, University of Guanajuato (UG) Noria Alta S/N 36050 Guanajuato Gto Mexico jebaez@ugto.mx; b Advanced Functional Materials & Nanotechnology Group, Centro de Investigación en Materiales Avanzados S. C. (CIMAV-Unidad Monterrey) Av. Alianza Norte 202, Autopista Monterrey-Aeropuerto Km 10, PIIT Apodaca-Nuevo León 66628 C.P. Mexico; c Instituto de Química Aplicada, Universidad del Papaloapan Circuito Central 200 Parque Industrial, San Juan Bautista Tuxtepec C. P. 68301 Oaxaca Mexico

## Abstract

A challenge in the design of biocompatible polymers involves the use of non-cytotoxic catalysts in the ring-opening polymerization (ROP) of lactones. In this study, a family of seven mono-, di-, and tricarboxylic acids was used as organocatalysts for the ROP of ε-caprolactone (CL) and l-lactide (l-LA). The use of a long-chain aliphatic alcohol such as 1-docosanol (C_22_OH) has been demonstrated to accelerate the polymerization rate. For the ROP of CL, carboxylic acids with p*k*_a_ values less than 4 and an increase in functional groups exhibited the highest catalytic efficiency. In contrast, the ROP of l-LA exhibited conversion that was independent of the number of carboxyl groups or p*k*_a_. Random copolymers of PCL-*co*-PLLA were successfully synthesized using citric acid as an organocatalyst and C_22_OH as an initiator. Increasing the l-LA content was observed to reduce the melting enthalpy without compromising semi-crystallinity, which is a behavior that is attributed to the docosyl terminal group.

## Abstract 2

Compounds derived from carboxylic acids were used as organocatalysts in the homopolymerization and copolymerization of lactones by the ring-opening polymerization (ROP) mechanism.

## Graphical abstract

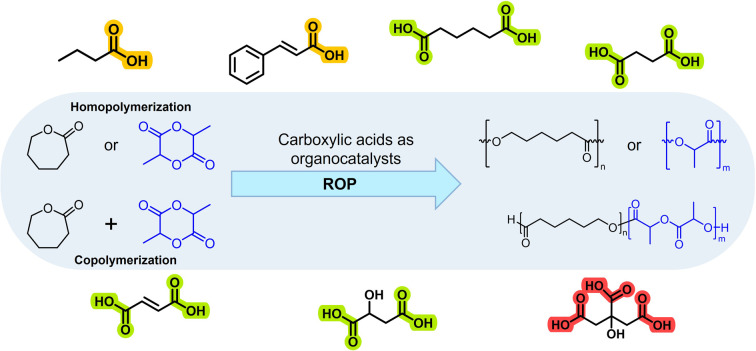

## Introduction

Ring-opening polymerization (ROP) has been the predominant method for synthesizing polyesters derived from lactones and lactides due to its capacity to control molecular weight and architecture.^1,2^ One of the most important reasons for synthesizing aliphatic polyesters is their biodegradability.^3–7^ In general, the ROP of lactones involves metal-based catalysts, including complexes based on Lewis acidic metals such as tin, aluminum, zinc, and molybdenum.^8^ These catalysts enable the preparation of polyesters with shorter reaction times and temperatures between 0 and 150 °C.

Polycaprolactone (PCL) and poly(l-lactide) (PLLA) are two of the most common polyesters and are synthesized by ROP of ε-caprolactone (CL) and l-lactide (l-LA), respectively. PCL is a semicrystalline, hydrophobic, and biodegradable polymer with a low melting temperature, flexibility, the ability to form blends, and a slow degradation rate. Its slow hydrolytic degradation rate compared to other aliphatic polyesters has enabled applications such as controlled drug-delivery systems,^9^ scaffolds for tissue engineering,^10^ and long-term implants.^11^ PLLA exhibits faster hydrolytic degradation, a high melting point, brittleness, and rigidity. Due to its extreme biocompatibility and faster degradation rate compared to PCL, it is applied in resorbable sutures,^12^ orthopedic fixation devices,^13^ and controlled-release matrices.^14^ To address the limitations of these polyesters, copolymerization of CL and l-LA has been demonstrated to effectively integrate the distinct advantages of PCL and PLLA, resulting in CL/LLA copolymers that are well suited for use in biomedical devices^15,16^ and biodegradable materials.^17^

Biomedical applications of PCL and PLLA require metal-free materials due to the potential for residual metals to remain in the polymer matrix, which may result in toxicological or cytotoxic effects.^18^ To address this challenge, novel catalysts have been explored, and one notable proposal involves the use organocatalysts in the ROP of lactones.

The use of organocatalysis in polymer science is a tool that has enabled chain- and step-growth polymerizations, as well as polymer functionalization.^19^ For example, polymerizations like organocatalyzed atom transfer radical polymerization (O-ATRP) can use cyclic macroinitiators to obtain grafted polymers.^20^

A classification system has been proposed for these organocatalysts and consists of three main groups. The first group comprises basic organocatalysts that are capable of subtracting a proton from the initiator (commonly aliphatic or aromatic alcohols) and have high values of p*k*_a_ (from 24 to 26 in acetonitrile), which facilitates the nucleophilic attack on the carbonylic carbon of the lactone. The most widely used basic organocatalysts in ROP are diazabicycloundecene (DBU),^21^ triazabicyclodecene (TBD),^22^ and methyltriazabicyclodecene (MTBD).^23^ The use of basic organocatalysts gives rise to transesterification side reactions as a negative aspect.

The second group comprises bifunctional systems of organocatalysts, which can achieve concomitant activation between the monomer and initiator and increase the polymerization rate. In the synthesis of some lactones, urea/base systems^24^ facilitate shorter reaction times, mild conditions and minimal side reactions. The third group is composed of acid organocatalysts, which have been shown to promote electrophilic activation of the monomer. In this process, the oxygen of the carbonyl is protonated and acts as an activated species that reacts easily with the initiator. These organocatalysts are the least studied due to disadvantages such as corrosive properties, hygroscopic tendencies, the fact that they are derived from strong acids, and their susceptibility to side reactions.

Brønsted acids have been used as catalysts in the ROP of lactones, including hydrochloric acid in diethyl ether (HCl·Et_2_O),^25,26^ methanesulfonic acid (MSA),^27,28^ trifluoromethanesulfonic acid (TfOH),^29^ 2,4-dinitrobenzenesulfonic acid (DNBA),^30^ diphenyl phosphate (DPP),^31^ phosphoramidic acid (PPA),^32^ imidodiphosphoric acid (IDPA),^33^ and carboxylic acids. Carboxylic acids are simple, but there has been limited exploration of this class of hydrogen-bond donors. Nevertheless, they can promote ROP and feature tunable acidity, biocompatibility, and availability, which makes them attractive candidates as sustainable organocatalysts. A number of carboxylic acids have been used in the ROP of lactones, including succinic acid,^34^ fumaric acid,^35^ tartaric acid,^36,37^ and benzoic acid.^38^

In contrast to phosphoric and phosphoramidic acids, which function as efficient Brønsted catalysts, operating mainly through proton shuttles in both the nucleophilic addition and ring-opening steps;^32^ the organocatalytic activity of carboxylic acids derives from localized hydrogen-bonding networks.^39^ This highlights the distinct yet complementary catalytic behavior of carboxylic acids, thereby extending the scope of green Brønsted acid catalysis for ROP. As can be observed in Table 1, carboxylic acids exhibit less extreme p*k*_a_ values, avoiding both the extremes of high and low p*k*_a_ values. The polydispersity values for all organocatalysts listed range from 1.04 to 1.97. Considering this range, it is preferable to use accessible and comparatively safer organocatalysts, such as carboxylic acids. While other non-metal catalysts exhibit more controlled acidity but reduced structural flexibility, the carboxylic acids evaluated in this study allowed modulation of acid strength and active site density through the number of carboxyl groups. This approach offers a simple, economical and potentially less hazardous route for ROP.

**Table 1:** Comparative analysis among a series of non-metal catalysts and their respective catalytic activities^a^

Non-metal catalyst	p*k*_a_^a^	Monomer	Reaction time (h)	Conversion rates	*Đ*_M_	Reference
DBU	13.5^b^	LA	1	43–99%	1.06–1.97	Sherck, 2016 [ref. 21]
		l-LA, δ-VL, ε-CL	1–120	78–99%	1.04–1.08	Lohmeijer, 2006 [ref. 23]
TBD	15.2^b^	LA	20 min–120 min	17–77%	1.09–1.25	Moins, 2020 [ref. 22]
		l-LA, δ-VL, ε-CL	20 s–8 h	52–99%	1.05–1.19	Lohmeijer, 2006 [ref. 23]
MTBD	15.0^b^	l-LA, δ-VL, ε-CL	0.5–120	78–99%	1.05–1.10	Lohmeijer, 2006 [ref. 23]
MSA	−1.9	ε-CL	1.5–2	98%	1.07	Gazeau-Bureau, 2008 [ref. 27]
		β-BL	1–26	96%	1.14–1.28	Couffin, 2013 [ref. 28]
TfOH	−14.9	ε-CL	1.5–7	98%	1.15–1.22	Gazeau-Bureau, 2008 [ref. 27]
		d,l-LA	2.5–28	>96%	1.13–1.48	Bourissou, 2005 [ref. 29]
DNBA	−2.2	ε-CL	4–20	45–97%	1.10–1.21	Wang, 2014 [ref. 30]
DPP	2.4	δ-VL, ε-CL	1–43	61–97%	1.06–1.09	Makiguchi, 2011 [ref. 31]
PPA	−3	ε-CL	0.3–5.5	98%	1.06–1.14	Delcroix, 2011 [ref. 32]
IDPA	11.5^c^	δ-VL, ε-CL	1.5–48	70–98%	1.12–1.24	Kan, 2013 [ref. 33]
Succinic acid	4.19	ε-CL	24	60–78%	1.40–1.48	Oledzka, 2010 [ref. 34]
Fumaric acid	3.02	ε-CL	24	64–95%	1.20–1.23	Oledzka, 2010 [ref. 34]
			6–24	61–91%	1.06–1.14	Sanda, 2001 [ref. 35]
Tartaric acid	2.98	ε-CL	24	>90%	1.49	Persson, 2006 [ref. 36]
		δ-VL, ε-CL	2–7	66–99%	1.3–1.35	Casas, 2004 [ref. 37]
Benzoic acid	4.2	ε-CL, l-LA	1.25–110	89–97%	1.09–1.41	Mezzasalma, 2018 [ref. 38]

a LA, l-lactide; δ-VL, δ-valerolactone; ε-CL, ε-caprolactone. ^a^Obtained from the cited reports. ^b^p*k*_a_ values in water. ^c^p*k*_a_ value in acetonitrile.

**Scheme 1:**
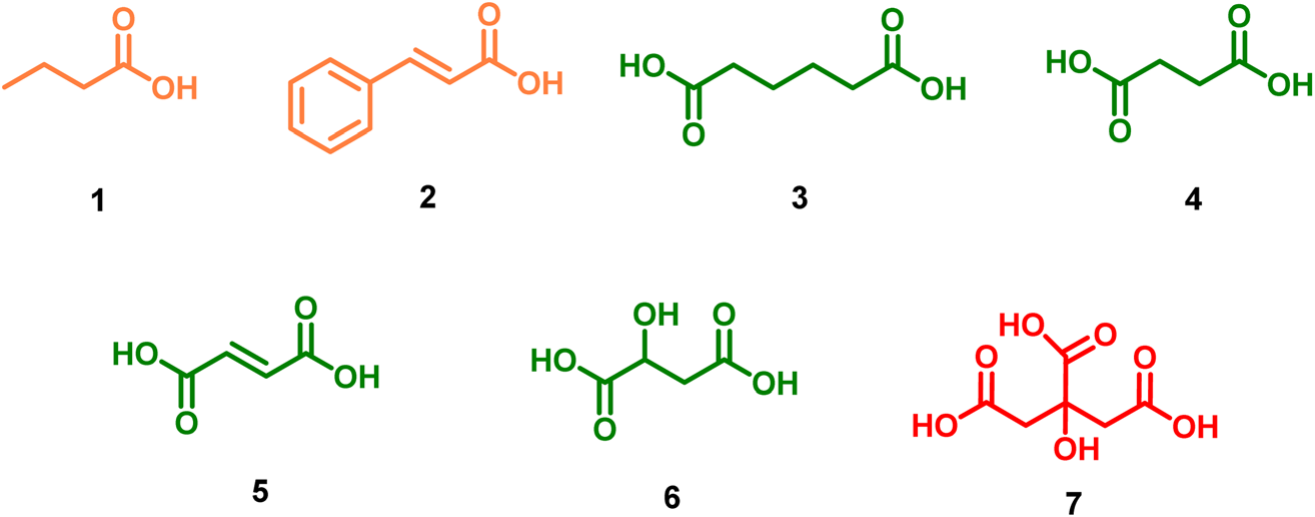
Structure of 1 butyric acid, 2 cinnamic acid, 3 adipic acid, 4 succinic acid, 5 fumaric acid, 6 malic acid and 7 citric acid, carboxylic acids used as organocatalysts.

This study presents a systematic evaluation of a family of carboxylic acid organocatalysts for the ROP of CL and l-LA, as well as their copolymerization. Mono-, di-, and tricarboxylic acids (Scheme 1) were compared to elucidate the role of acidity and functional groups' multiplicity in the catalytic activity and polymer microstructure. This approach provides new insights into the design of metal-free catalytic systems for the synthesis of biodegradable polyesters.

## Experimental

### Materials

ε-Caprolactone (CL) was supplied by Aldrich Chemical Co., dried over calcium hydride (CaH_2_) for 24 h; distilled under nitrogen atmosphere (N_2_) and reduced pressure before use. l-Lactide (l-LA), 1-docosanol (C_22_OH), *n*-heptanol, butyric acid (p*k*_a_ = 4.82),^40^ cinnamic acid (p*k*_a_ = 4.46),^41^ succinic acid (p*k*_a_ = 4.19),^42^ fumaric acid (p*k*_a_ = 3.02),^43^ malic acid (p*k*_a_ = 3.46),^44^ adipic acid (p*k*_a_ = 4.43),^45^ citric acid (p*k*_a_ = 3.13),^46^ levulinic acid (p*k*_a_ = 4.64),^47^ and tricarballylic acid (p*k*_a_ = 3.78)^48^ were purchased from Aldrich Chemical Co. and used without further purification.

### Synthesis of α-hydroxy-ω-docosyl homopolymers (PCL and PLLA)

**Scheme 2:**
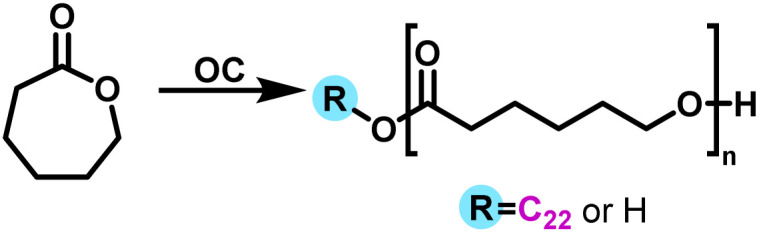
General synthesis of PCL by ROP with or without 1-docosanol as initiator and using organocatalysis where OC = 1, 2, 3, 4, 5, 6 or 7.

**Scheme 3:**
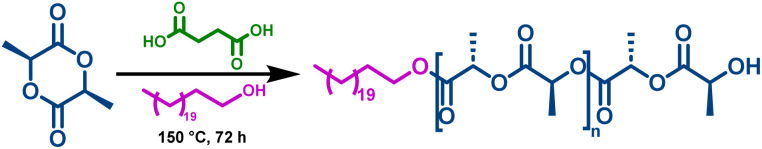
ROP of l-LA using 1-docosanol as initiator and succinic acid 4 as organocatalyst.

In a dried 20 mL vial with hermetic cap, ε-caprolactone (CL) (10 mmol, 1.14 g), 1-docosanol (1 mmol, 0.326 g) and the organocatalysts (0.1 mmol, molar ratio [[*M*]_0_/[*I*]_0_/[Cat]_0_ = 100/10/1] were added and heated in an aluminium block at 150 °C for 4 h and stirred at 250 rpm (Scheme 2). Conversion and number-average molecular weight (*M*_*n*_) were monitored by ^1^H NMR. The polymer obtained was dissolved in chloroform (CHCl_3_), precipitated in cold methanol, recovered by filtration, and left to dry at room temperature for one night. *M*_*n*_ was calculated by ^1^H NMR with end group analysis. A similar methodology was used for the synthesis of α-hydroxy-ω-docosyl PLLA homopolymer (Scheme 3).

*M*_*n*_ (calcd) = 1,468, *M*_*n*_ (NMR) = 1290 (Conv. = 98%), *M*_*n*_ (GPC) = 3015, *Đ*_M_ = 1.22. IR (cm^−1^) 3442 (*ν*, OH, PCL), 2945 (*ν*_as_, CH_2_, PCL), 1721 (*ν*, C=O, PCL), 1161 (*ν*_as_, C–(C=O)–O, PCL)). NMR data for α-hydroxy-ω-docosyl PCL homopolymer. ^1^H NMR (500 MHz, CDCl_3_, ppm): *δ* 4.17 [–(C=O)–CH_2_–CH_2_–CH_2_–CH_2_–*CH*_2_–O–(C=O)–CH_2_–C(COOH)(OH)–CH_2_–(C=O)–OH, PCL], 4.06 [CH_3_–(CH_2_)_21_–O–(C=O)–CH_2_–CH_2_–CH_2_–CH_2_–*CH*_2_–O–(C=O)–, PCL and CH_3_–(CH_2_)_19_–CH_2_–*CH*_2_–O–, 1-docosanol], 3.64 [–(C=O)–CH_2_–CH_2_–CH_2_–CH_2_–*CH*_2_–OH, PCL], 2.85 [–O–(C=O)–*CH*_2_–C(COOH)(OH)–*CH*_2_–(C=O)–OH, citric acid], 2.30 [–(C=O)–*CH*_2_–CH_2_–CH_2_–CH_2_–CH_2_–O–, PCL, 1.64 [–(C=O)–CH_2_–*CH*_2_–CH_2_–*CH*_2_–CH_2_–O–, PCL], 1.58 [CH_3_–(CH_2_)_19_–*CH*_2_–CH_2_–O–, 1-docosanol], 1.40 [–(C=O)–CH_2_–CH_2_–*CH*_2_–CH_2_–CH_2_–O–, PCL], 1.25 [CH_3_–(*CH*_2_)_19_–CH_2_–CH_2_–O–, 1-docosanol], 0.87 [*CH*_3_–(CH_2_)_19_–CH_2_–CH_2_–O–, 1-docosanol].

### Synthesis of PCL-PLLA copolymer

In a dried 20 mL vial with hermetic cap, ε-caprolactone (CL) (9 mmol, 1.03 g), l-lactide (l-LA) (1 mmol, 0.144 g), 1-docosanol (1 mmol, 0.326 g) and citric acid as organocatalyst (0.1 mmol, molar ratio ([*M*]_0 CL_ + [*M*]_0 L–LA_)/[*I*]_0_/[Cat]_0_ = 100/10/1] were added and heated in an aluminium block at 150 °C for 24 h and stirred at 250 rpm. Conversion was monitored by ^1^H NMR. The polymer obtained was dissolved in chloroform (CHCl_3_), precipitated in cold methanol, recovered by filtration, and left to dry at room temperature for one night. Different molar ratios of CL and l-LA were used, always maintaining an initial proportion of 10 mmol (example: for a CL/l-LA ratio = 80%/20%, 8 mmol of CL and 2 mmol of l-LA were added).

The average lengths of the lactidyl unit (L_LA_ = LLL) and the caproyl unit (L_C_) were determined from the ^13^C NMR spectrum (acquisition time: 18 h) using: L_LL_ = [LLLLLL + LLLLC + CLLLL + CLLC]/[CLLC + ½(LLLLC + CLLLL)], and L_C_ = [LLCLL + CCLL + LLCC + CCC]/[ LLCLL + ½(CCLL + LLCC)], where LLLLLL, LLLLC, CLLLL, CLLC, LLCLL, CCLL, LLCC, and CCC correspond to the integrated values of signals from triad sequences.^49–51^

IR (cm^−1^) 3516 (*ν*, OH, PLLA), 2938 (*ν*_as_, CH_2_, PCL), 1747 (*ν*, C=O, PCL and PLLA), 1443 (*δ*_as_, CH_3_, PLLA), 1167 (*ν*_as_, C–(C=O)–O, PCL), 1044 (*ν*_as_, O–C–C, PCL), 733 (*ρ*, CH_2_, PCL). NMR data for PCL-*co*-PLLA copolymer. ^1^H NMR (500 MHz, CDCl_3_, ppm): *δ* 5.13 [–(C=O)–*CH*(CH_3_)–O–, PLLA attached to PLLA and [–(C=O)–*CH*(CH_3_)–O–(C=O)–(CH_2_)_5_–O–, PLLA attached to PCL], 4.32 [–*CH*(CH_3_)–OH, PLLA], 4.24 [*CH*_22_–O–CO–CH(CH_3_)–O–, 1-docosanol attached to l-LA], 4.10 [–(C=O)–(CH_2_)_4_–*CH*_2_–O–(C=O)–CH(CH_3_)–O, PCL attached to PLLA], 3.61 [–(C=O)–(CH_2_)_4_–*CH*_2_–OH, PCL], 2.38 [(C=O)–CH(CH_3_)–O–(C=O)–*CH*_2_–(CH_2_)_4_–O–, PLLA attached to PCL], 2.27 [–(C=O)–*CH*_2_–CH_2_–CH_2_–CH_2_–CH_2_–O–, PCL], 1.63 [–(C=O)–CH_2_–*CH*_2_–CH_2_–*CH*_2_–CH_2_–O–, PCL], 1.58 [CH_3_–(CH_2_)_19_–*CH*_2_–CH_2_–O–, 1-docosanol and –(C=O)–CH(*CH*_3_)–O–, PLLA], 1.38 [–CO–CH_2_–CH_2_–*CH*_2_–CH_2_–CH_2_–O–, PCL], 1.23 [CH_3_–(*CH*_2_)_19_–CH_2_–CH_2_–O–, 1-docosanol], 0.86 [*CH*_3_–(CH_2_)_19_–CH_2_–CH_2_–O–, 1-docosanol].

### Characterization methods

*Nuclear Magnetic Resonance* (*NMR*): ^1^H and ^13^C NMR were recorded at room temperature on Bruker Avance III HD 500 MHz (500 MHz ^1^H and 125 MHz ^13^C). CDCl_3_ or DMSO-*d*_6_ were used as solvents, and all spectra were referenced to the residual solvent CDCl_3_ [*δ* (ppm) 7.26 (^1^H) and 77.0 (^13^C)] or [*δ* (ppm) 2.50 (^1^H) and 39.52 (^13^C)]. *Fourier Transform Infrared Spectroscopy* (*FT-IR*): all samples were recorded with an attenuated total reflectance spectroscopy (ATR) accessory in a PerkinElmer Spectrum Two FT-IR spectrometer. *Differential Scanning Calorimetry* (*DSC*): thermograms were performed in a Q200 TA instrument. Three scans were obtained with two heating (25–130 °C and −30–130 °C) and one cooling (130–−30 °C) between them, at a rate of 10 °C min^−1^ and under a nitrogen purge. *Gel permeation chromatography* (*GPC*): polyester samples were dissolved in THF (5 mg/5 mL) and heated at 37 °C for one hour before filtration. GPC measurements were determined using an Agilent Technologies PL-GPC 220 Gel Permeation chromatograph, with PLgel 5 μm MIXED-D Columns to elute samples at the flow rate of 1 mL min^−1^ HPLC grade tetrahydrofuran (THF). Polystyrene standards (Polymer Laboratories) were used for calibration. *Polarized Optical Microscopy* (*POM*): POM micrographs were obtained using a Nikon light Eclipse E200 microscope, photographs were taken using iPhone 13 mini. The samples were mounted on glass slides as a thin film melted at 100 °C and applying manual pressure between the two slides containing the sample and cover glass. The samples were cooled at room temperature before analysis. Samples were collected with a magnification of 40×. *Matrix-Assisted Laser Desorption Ionization Time-of Flight* (*MALDI-TOF*): MALDI-TOF spectra were recorded in the reflector mode by using a Bruker Microflex instrument that incorporates a nitrogen laser with a wavelength of 337 nm in positive polarity accelerated at 20 kV. The preparation of the samples and the matrix (dithranol) were carried out by their dissolution in THF and water/acetonitrile/trifluoroacetic acid solution (60/40/0.1), respectively. Then, 2 mL of the sample solution were mixed with 5 mL of the matrix solution. From the resulting mixture, 2 mL was taken and placed in a stainless-steel plate for 20 minutes to evaporate the solvent. Reserpine, angiotensin II, melittin and insulin were used for the calibration.

## Results and discussion

### Synthesis of poly(ε-caprolactone) (PCL) by succinic acid as organocatalyst

Seven carboxylic acids (Scheme 1) were tested as organocatalysts for ROP of CL in bulk conditions (Scheme 2). To optimize the reaction conditions and compare the seven organocatalysts, the amount of catalyst and temperature were examined as reaction parameters while using succinic acid 4 as the model organocatalyst. This was motivated by reports of succinic acid promoting ROP of CL.^52^ To determine the optimal conditions for both parameters, the conversion was quantified by ^1^H NMR.

**Fig. 1:**
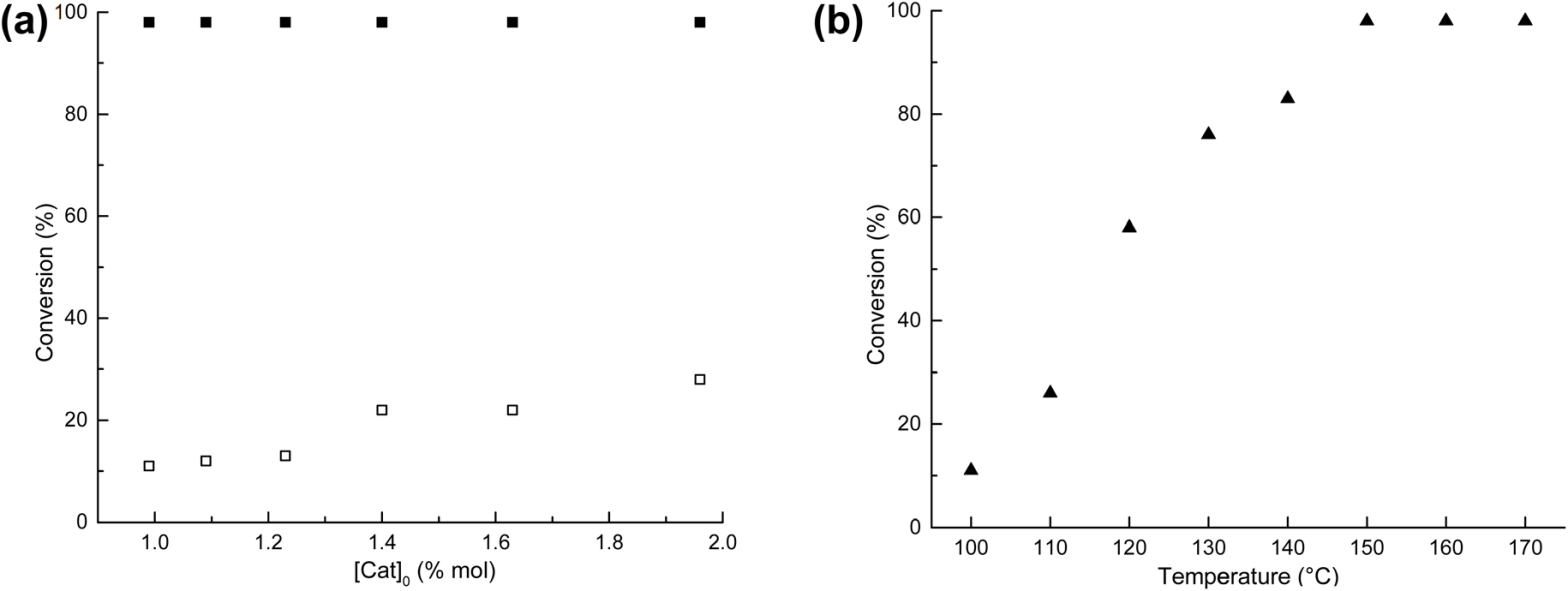
ROP of ε-CL ([CL]_0_ = 10 mmol) catalyzed by 4 succinic acid: (a) effect of the amount of catalysts used at 100 °C (open square) and 150 °C (filled square), and (b) effect of temperature ([*M*]_0_/[Cat]_0_ = 100, 24 h (from 100–140 °C), 12 h (150 °C), 8 h (160 °C), and 7 h (170 °C)).

The first parameter evaluated was the amount of catalyst (succinic acid 4). The reactions were carried out in bulk conditions at 100 °C and 150 °C for 24 hours with variation of the initial ratio of monomer (ε-CL) to organocatalyst ([*M*]_0_/[Cat]_0_). As demonstrated in Fig. 1a, the reactions conducted at 150 °C exhibited conversions exceeding 98%, even with the lowest amount catalytic ([*M*]_0_/[Cat]_0_ = 100; 0.1 mmol).

A gradual increase of conversion occurred as the temperature increased from 100 °C to 150 °C, and the optimum temperature was found to be 150 °C (Fig. 1b). When the reaction was carried out at this temperature, the highest conversion was achieved at 12 hours (>98%). At 160 °C and 170 °C, the maximum conversion was obtained after 8 and 7 hours, respectively. Thus, the optimal conditions for the ROP of ε-CL in the presence of succinic acid 4 without initiator were ultimately found to be *T* = 150 °C and [*M*]_0_/[Cat]_0_ = 100 (0.1 mmol of organocatalyst) at 12 h.

**Fig. 2:**
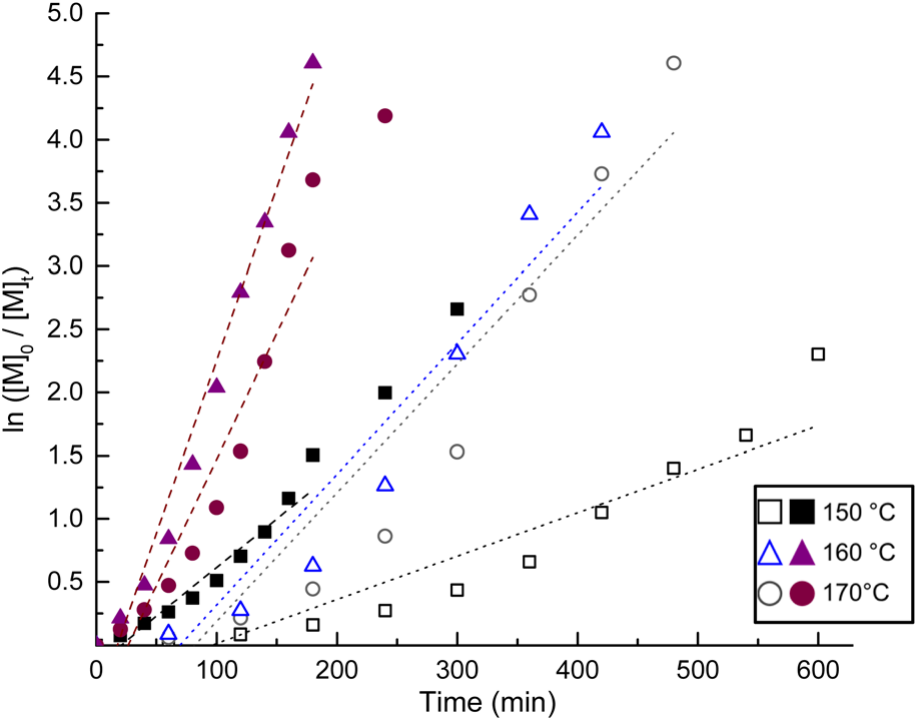
Semilogarithmic graphics for the ROP of ε-CL catalyzed by 4 succinic acid against the reaction time at different temperatures for (a) without initiator (open figures): *k* = 6.16 × 10^−5^ s^−1^ for 150 °C; *k* = 1.65 × 10^−4^ s^−1^ for 160 °C; *k* = 1.71 × 10^−4^ s^−1^ for 170 °C; and (b) with 1-docosanol as initiator (filled figures): *k* = 1.31 × 10^−4^ s^−1^ for 150 °C; *k* = 3.41 × 10^−4^ s^−1^ for 160 °C; *k* = 4.5 × 10^−4^ s^−1^ for 170 °C.

Six experiments were conducted to examine the kinetics of the ROP of ε-CL catalyzed by succinic acid with and without 1-docosanol as initiator. The temperature was varied from 150 to 170 °C. The profile of the kinetics in Fig. 2 demonstrates good concordance with a linear dependency, which suggests a pseudo-first-order reaction with respect to the monomer. A proportional dependency on the consumption of ε-CL was detected as the temperature increased from 150 to 170 °C. The incorporation of 1-docosanol as the initiator resulted in an increase of almost threefold in the conversion rate of the monomer (ε-CL) (Fig. 2). The results indicated that the optimal conditions for the ROP of ε-CL in the presence of succinic acid 4 and 1-docosanol as the initiator were *T* = 150 °C and [*M*]_0_/[*I*]_0_/[Cat]_0_ = 100/10/1 (10 mmol of monomer, 1 mmol of initiator, and 0.1 mmol of organocatalyst) for a duration of 4 h.

### Synthesis of poly(ε-caprolactone) (PCL) by a family of carboxylic acids as organocatalysts

Once the optimal conditions for the ROP of ε-CL were established, ROP reactions with the rest of the carboxylic acids as organocatalysts were performed with the conditions established for succinic acid ([*M*]_0_/[*I*]_0_/[Cat]_0_ = 100/10/1; *T* = 150 °C; *t* = 4 h). Three of the carboxylic acids used as organocatalysts (Table 2, entries 5, 6, and 7) exhibited quantitative conversions with reduced reaction times in comparison to succinic acid (Table 2, entry 4). The remaining carboxylic acids [butyric acid 1, cinnamic acid 2, and adipic acid 3 (entries 1–3)] achieved conversions less than 90%, and butyric acid 1 was the least active catalyst with only 45% conversion (Table 2, entry 1).

**Table 2:** Ring-opening polymerization (ROP) of ε-CL using different organocatalysts^a^

Entry	OC	Time (min)	[*M*]_0_/[*I*]_0_/[Cat]_0_	Conv^c^ (%)	DP_(NMR)_^c^	*M*_*n* NMR_ c (g mol^−1^)	*M*_*n* GPC_ d (g mol^−1^)	*Đ*_M_ d
1	1	240	100 : 10 : 1	45	2.8	650	1810	1.70
2	2	240	100 : 10 : 1	88	6.7	1090	2230	1.44
3	3	240	100 : 10 : 1	85	6.6	1080	2190	1.49
4	4	240	100 : 10 : 1	>98	8.5	1380	2420	1.50
5	5	220	100 : 10 : 1	>98	7.9	1230	2240	1.43
6	6	180	100 : 10 : 1	>98	8.2	1260	2260	1.48
7	7	100	100 : 10 : 1	>98	8.7	1320	2420	1.56
8	**bSn(Oct)**_**2**_	25	100 : 10 : 1	>98	6.8	1110	2990	1.34
9	8	240	100 : 10 : 1	60	3.9	770	2390	1.40
10	9	180	100 : 10 : 1	>98	7.6	1196	2710	1.46

a Bulk ROP of ε-CL with 1-docosanol as initiator at 150 °C, ratio [*M*]_0_/[*I*]_0_/[Cat]_0_ = 100 : 10 : 1 (mmolar ratio = 10 : 1 : 0.1).

b Metal-based catalyst.

c Determined by ^1^H NMR.

d Determined by GPC.

**Fig. 3:**
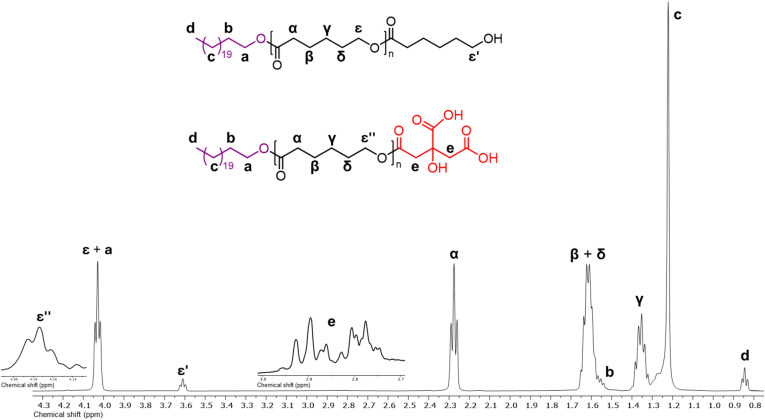
^1^H NMR spectrum at room temperature in CDCl_3_ of α-hydroxyl-ω-docosyl-PCL obtained using 7 citric acid as organocatalyst (Table 1, entry 7).

The chemical nature of the PCL homopolymers was determined by NMR. As an example, Fig. 3 shows the ^1^H NMR spectrum of α-hydroxyl-ω-docosyl-PCL obtained using citric acid as the organocatalyst (Table 2, entry 7). The spectrum shows the characteristic peaks of the repeat unit attributed to the methylenes of the main chain of the PCL [–CH_2_–O–, 4.06 ppm (*ε*), and –CH_2_–(C=O)–O–, 2.30 ppm (α)], as well as the signal for the PCL hydroxyl terminal group [CH_2_–OH, 3.64 ppm (ε′)]. The incorporation of the 1-docosanol to the PCL chain was evidenced by the signals of the methylenes of the docosyl main chain [–CH_2_–, 1.25 ppm (c)], methylene attached to the PCL chain [–CH_2_–O–PCL, 4.06 ppm (a)], and the terminal methyl [CH_3_–, 0.87 ppm (d)]. Additionally, signals of methylenes from citric acid 7 were detected [–CH_2_–C(OH)–CH_2_, 2.80 ppm (e)], which suggests that it was inserted at the end of the PCL chain.

**Fig. 4:**
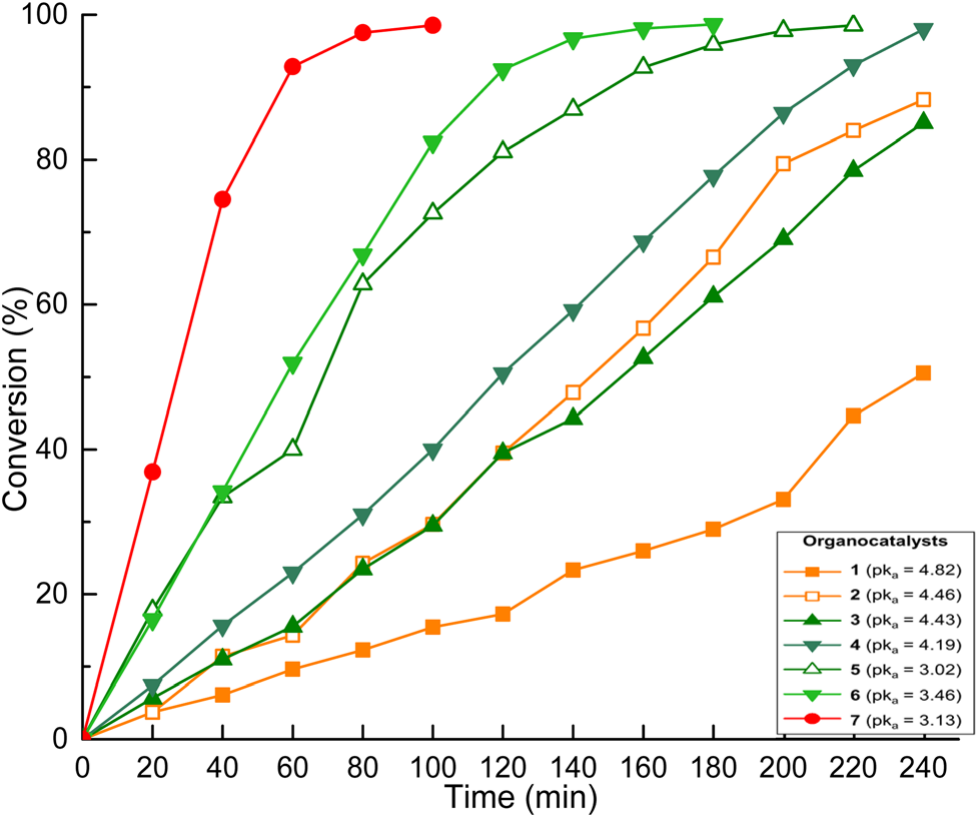
Comparison of the conversion rate at 150 °C of the different organocatalysts.

Comparison of the conversion rates revealed the efficacy of citric acid 7, which achieved 98% conversion at 100 minutes (Fig. 4, red circle). Fig. 4 shows that there was a decrease in organocatalytic activity as the p*k*_a_ value increased, with the most active molecules being those with a p*k*_a_ value less than 3.5. Carboxylic acids function as Brønsted acids by protonating the exocyclic oxygen of CL. Thus, a decrease in the acidity of these molecules corresponded to a reduction in the dissociation capacity of the acid proton. Therefore, there was a decrease in the activation of the monomer, which is consistent with prior observations of the behavior of organic acids as organocatalysts in the ROP of the CL.^34,53^

The time required to complete the polymerization showed no direct correlation with the acidity of the most active molecules (citric acid 7, malic acid 6, and fumaric acid 5) (Fig. 4). Structural analysis of the molecules revealed that organocatalytic activity was associated with an increase in carboxylic acid (–COOH) functionalities present in the chemical structure of the organocatalyst. For example, triprotic acid (citric acid 7) was more active than diacids (malic acid 6, fumaric acid 5, *etc.*), which exhibited greater activity than monofunctional acids (butyric acid 1 and cinnamic acid 2) (Scheme 1). A linear relationship was observed between the molecular weight and the conversion percentage (Fig. S3).

The diacid molecules include three molecules with similar structures (succinic 4, fumaric 5, and malic 6 acids) and one molecule with a longer chain in its structure (adipic acid 3) (Scheme 1, orange structures). Increased size of the alkyl group of the organocatalyst was associated with a decrease in the acidity constant (p*k*_a_ (6) = 3.46 to p*k*_a_ (3) = 4.43). As previously established, a lower p*k*_a_ resulted in greater organocatalytic activity; therefore, shorter distance between the functionalities (reduction in the length of the alkyl group) results in greater organocatalytic activity. A notable tendency related to the chemical structure was the increase in the organocatalytic activity with the increase in the number of functional groups in the carbon skeleton of the organocatalyst. The conversion was lowest for succinic acid, followed by fumaric acid, malic acid, and citric acid.

**Scheme 4:**
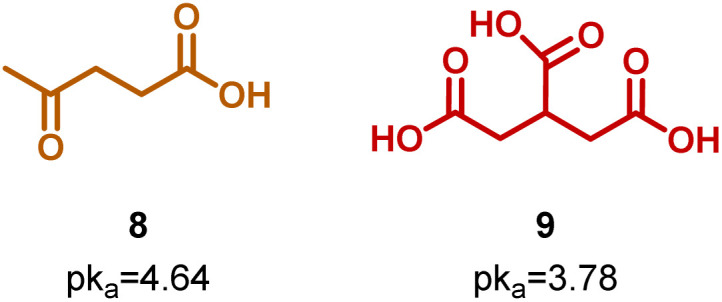
Structure of 8 levulinic acid, and 9 tricarballylic acid.

**Fig. 5:**
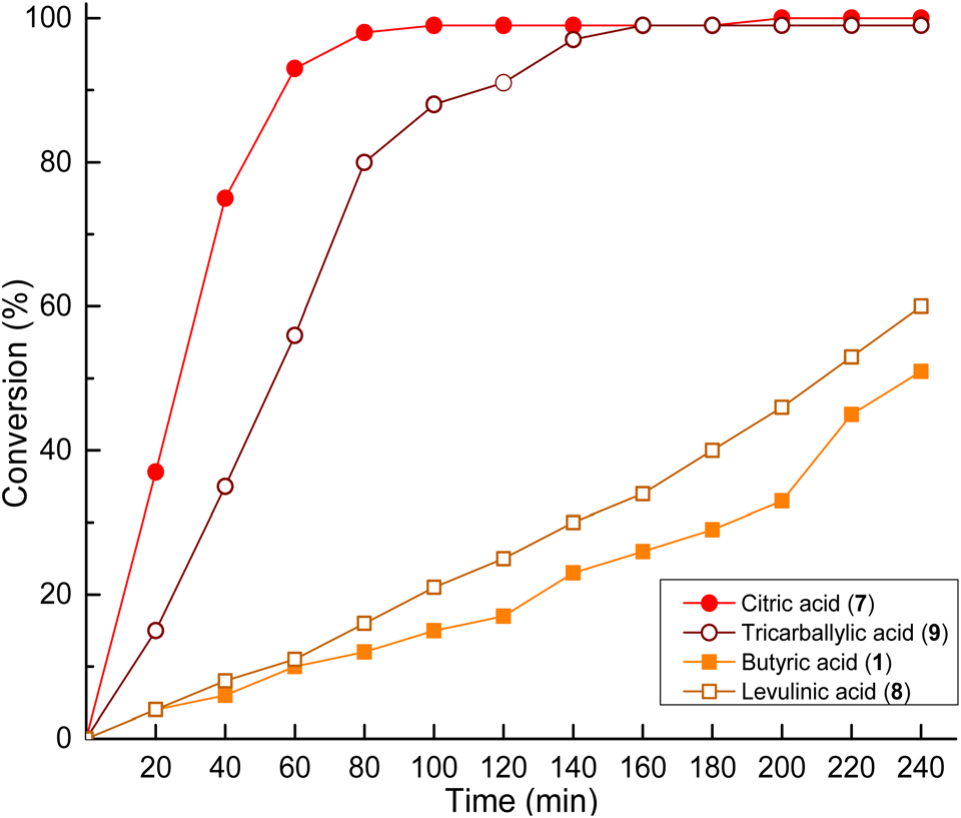
Comparison of the conversion rate at 150 °C of molecules analogous to citric and butyric acids.

Both acidity and the increase in functional groups were important factors to consider to select molecules with good performance as organocatalysts. To validate this perspective, a pair of molecules analogous to butyric acid 1 (C_3_H_7_CO_2_H) and citric acid 7 [HOC_3_H_4_(CO_2_H)_3_] were evaluated as organocatalysts for the ROP of CL: levulinic acid 8 (OC_4_H_7_CO_2_H) and tricarballylic acid 9 [C_3_H_5_(CO_2_H)_3_], respectively (Scheme 4 and Table 2, entry 9 and 10). Fig. 5 shows that there was a reduction in organocatalytic activity for tricarballylic acid 9 in comparison to citric acid 7, which is attributable to the absence of a hydroxyl group in its chemical structure. The conversion curve resembled the behavior of malic acid 6, despite the fact that malic acid 6 is more acidic (p*k*_a_ = 3.46) than tricarballylic acid 9 (p*k*_a_ = 3.78).

In the case of levulinic acid 8, a ketone-type functionality appears compared to butyric acid 1. The results showed a slight increase in the organocatalytic activity in comparison to the analogous butyric acid 1. This supports the significance of the hydroxyl-type functionality in the structure of organocatalysts and underscores the importance of the acidity of the molecules, with higher efficacy observed for organocatalysts with p*k*_a_ values less than 4, as discussed previously.

The catalytic efficiency was found to be directly related to both the acidity and number of carboxylic acid groups present in the organocatalyst. Acids with p*k*_a_ values below 4, like citric acid 7 and tricarballylic acid 9, produced higher conversion rates compared to less acidic monocarboxylic acids such as butyric acid 1 (Fig. 5). This effect can be explained by the formation of hydrogen-bonding interactions between the acid and both the initiator and monomer. The structure–activity relationship showed that functional group density had a stronger influence on kinetics than acid strength by itself. This was an important difference between carboxylic acids and phosphorus-based Brønsted acids, where proton-shuttling is typically observed.^32^ Multicarboxylic acids have been shown to promote networked hydrogen-bonding interactions, thereby accelerating monomer activation and propagation.^39^

Despite the predominant focus on organocatalysis as an area of research, organometallic catalysts maintain a significant role in the preparation of polymeric compounds. Tin(ii) 2-ethylhexanoate (Sn(Oct)_2_) is one of the most widely used compounds in the ROP of different lactones. To compare carboxylic acids as organocatalysts with a metallic system, ROP reaction for CL was performed using Sn(Oct)_2_ as a catalyst and compared to citric acid 7 as an organocatalyst. The Sn(Oct)_2_-catalyzed system exhibited first-order behavior with respect to the monomer. A value of *k* = 3.1 × 10^−3^ s^−1^ was obtained, which indicated that the reaction was almost 4 times faster when Sn(Oct)_2_ was used in comparison to citric acid (*k* = 7.9 × 10^−4^ s^−1^) (Fig. S2). This behavior was consistent with the established mechanism of Sn(Oct)_2_-catalyzed polymerizations, which have been reported to proceed through the formation of an alkoxide, a more nucleophilic species than neutral alcohols.

**Scheme 5:**
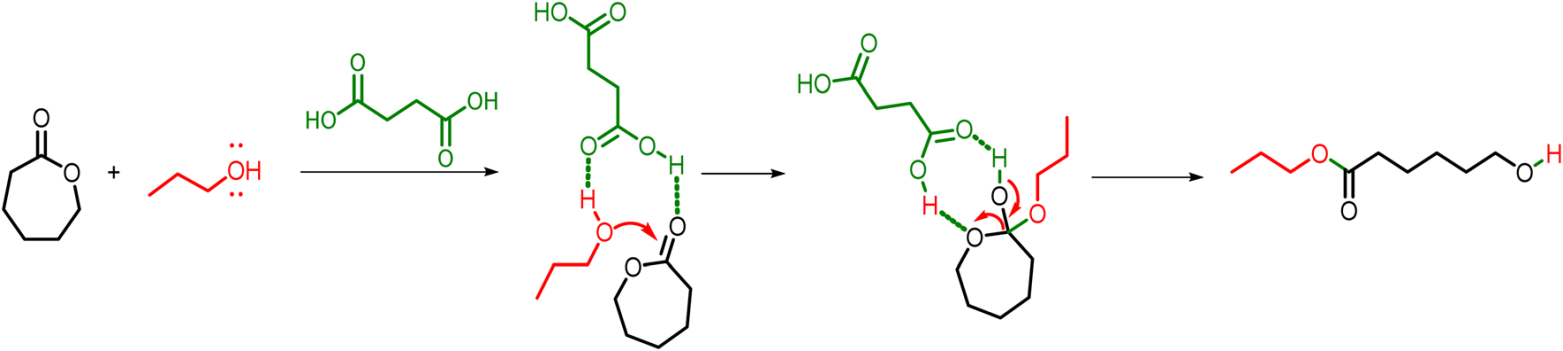
Possible bifunctional mechanism of the ROP of CL using 4 succinic acid as an organocatalyst.

As discussed previously, carboxylic acid activity in the ROP of lactones is attributed to the formation of adducts between the acid proton of the organocatalyst and the exocyclic oxygen of the lactone (activated monomer mechanism). The chemical structure of the organocatalyst determines the mechanism through which it functions, with the potential to act through a bifunctional mechanism.^32^Scheme 5 exhibits a proposal for a bifunctional mechanism involving carboxylic acid and alcohol concomitant interaction with the lactone. In the first step, the carbonyl group of the lactone is activated through hydrogen bonding interactions with succinic acid (carboxylic acid organocatalyst). This activation increases the electrophilicity of the carbonyl carbon and facilitates the nucleophilic attack of the alcohol (initiator).

This attack generates a tetrahedral intermediate, which is stabilized by proton transfer processes that are mediated by the acid catalyst. Subsequent collapse of the intermediate results in scission of the ester bond within the lactone ring, which leads to ring opening. The final product of this transformation is the linear ester.

Differential scanning calorimetry (DSC) was performed to analyze the thermal properties of PCL-derived oligomers. A recurring pattern of melting temperatures (*T*_m_) was observed with values ranging from 45 to 48 °C. The lowest *T*_m_ was observed for PCL homopolymers with the lowest conversion rates (Table S1). These low *T*_m_ values were attributed to the formation of oligomers.

**Fig. 6:**
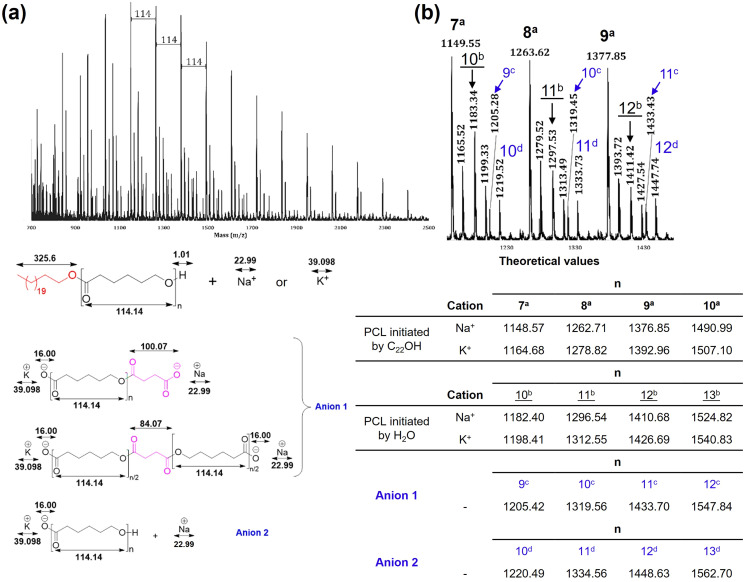
MALDI-TOF spectra (reflector mode)-hydroxyl-ω-docosyl-PCL. Where the numbers correspond to the DP of ε-caprolactone (CL) [–CO(CH_2_)_5_O–]_*n*_ units in the homopolymer initiated with C_22_OH (bold) and initiated with water (underlined). (a) Complete view from 700–2500 *m*/*z* fragments. (b) Expanded view from 1100–1480 *m*/*z* fragments doped with Na^+^. PCL initiated by H_2_O is illustrated in Scheme S1 (see SI).

Matrix-assisted laser desorption ionization time-of-flight (MALDI-TOF) analysis was used to visualize repeating units and terminal groups and confirm the chemical nature of the PCL homopolymer. Fig. 6 presents the MALDI-TOF spectrum of α-hydroxyl-ω-docosyl-PCL (Table 2, entry 4). The spectrum exhibited a characteristic curve that corresponded to the molecular weight distribution of a PCL oligomer. Furthermore, it showed signals that were separated by 114 mass units, which corresponded to the molecular weight of the CL monomer. The theoretical values of *M*_*n*_ were calculated with the following formula: *M* = 326.61 (*M*_w_ of C_22_OH) + DP_PCL_ × 114.14 (*M*_w_ of ε-CL) + 23 (Na^+^) or *M* = 326.61 (*M*_w_ of C_22_OH) + DP_PCL_ × 114.14 (*M*_w_ of ε-CL) + 39 (K^+^). For α-hydroxyl-ω-docosyl-PCL (Table 2, entry 4) DP corresponded to 8.5, considering rounded DP values of 9 resulted in theoretical values of 1376.8 (with Na^+^) or 1392.9 (with K^+^). This being similar and consistent with the values labeled as 9^a^ at the top of Fig. 6b, and to *M*_*n*_ obtained by NMR of Table 2 (1380 g mol^−1^). *M*_*n*_ GPC values (Table 2) being approximately double the value of *M*_*n*_ (NMR). This is relatively common due to the polystyrene standards used in the calibration curve.

An expanded view of Fig. 6a is shown in Fig. 6b. More intense fragments are indicated by bold numbers at the top of the MALDI-TOF curve, which corresponds to a degree of polymerization (DP) of 7 to 9 (doped with Na^+^) of the PCL initiated with 1-docosanol. The fragments indicated with underlined numbers correspond to DP from 10 to 12 (doped with Na^+^) of the PCL initiated with water present in the reaction media. Two other types of anions were observed through MALDI-TOF in addition to PCL initiated with 1-docosanol and PCL initiated with water. Anion 1 was consistent with a molecule of succinic acid that was either attached at the end of the PCL chain or positioned between two PCL molecules. Conversely, anion 2 was exclusive to the PCL chain, as indicated in Fig. 6.

It is common the presence of discrepancies of less than 1 Da between the labels in Fig. 6b and the corresponding data in the table of theorical values below. These differences are attributed to analyzer resolution and calibration accuracy.^54–56^ This does not affect the correct assignment of fragments or structural interpretation.

### ROP of l-LA using three different organocatalysts derived from carboxylic acids

Prior to the first exploration of copolymerization reactions, the efficiency of the organocatalysts in the homopolymerization of l-LA was evaluated. ROP of l-LA was conducted using the same conditions as those of CL ([*M*]_0_/[*I*]_0_/[Cat]_0_ = 100/10/1; *T* = 150 °C). Organocatalysts butyric acid 1, succinic 4, and citric acid 7 were used to determine whether the same pattern was appliable (Table 3).

**Table 3:** Ring-opening polymerization (ROP) of l-LA using three of the organocatalysts^a^

Entry	Time (h)	Conversion^b^ (%)		
		7	4	1
1	12	41	41	41
2	24	79	81	70
3	36	88	92	90
4	48	97	96	93
5	60	97	97	96
6	72	98	98	97

a Bulk ROP of l-LA with 1-docosanol as initiator at 150 °C, ratio [*M*]^0^/[*I*]^0^/[Cat]^0^ = 100 : 10 : 1 (mmolar ratio = 10 : 1 : 0.1). Organocatalysts used: 7 citric acid, 4 succinic acid, and 1 butyric acid.

b Determined by ^1^H NMR.

After 4 hours of reaction, the conversion was only a little more than 10%, so the reaction was allowed to occur for 12 hours. The reaction times were also extended to 72 hours to obtain conversion comparable to that for CL (98–99%).

**Fig. 7:**
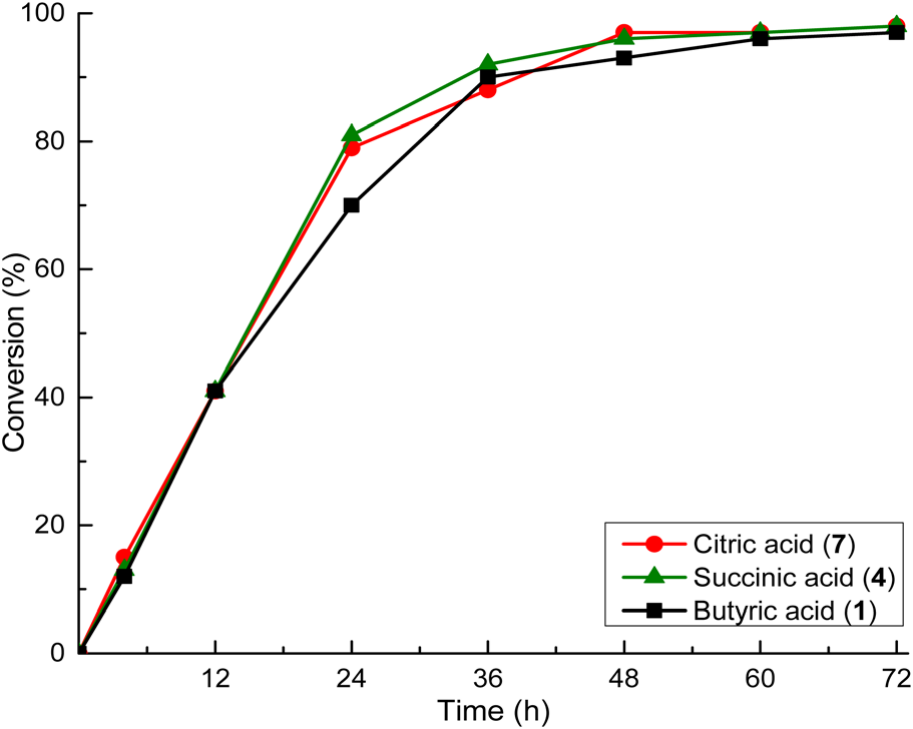
Conversion (%) *versus* time (h) for the ROP of l-LA using three different carboxylic acids as organocatalysts.

In the case l-LA, the organocatalytic activity was independent of the chemical structure, the number of functionalities, and the presence of additional functional groups of the organocatalyst. In contrast, the kinetics of polymerization was characterized by overlapping results, as shown in Fig. 7.

For ε-CL, carboxylic acids with p*k*_a_ < 4 and organocatalysts with multiple functional groups increase the electrophilic activation of the carbonyl group.^32^ In contrast, ROP of l-LA exhibited a weaker dependence on p*k*_a_ and catalysts functionality. This could be attributed to the steric rigidity of the lactide ring, and the steric hindrance of the methyl group at the propagating chain end.^57^ The interaction between the organocatalytic molecules such as carboxylic acids and CL was favored due to the absence of a methyl branch in the CL in comparison with l-LA. l-LA interaction with the organocatalytic species was at a relative long distance due to the steric hindrance (just providing protons in terms of Brønsted acid), where the same kinetic behavior in the ROP of l-LA was observed. The nucleophilic attack for the ROP of l-LA may be restricted by steric hindrance of the methyl group in the α-position of the carbonyl of the monomer. In this sense, the organocatalysis system also can cause side reactions, like epimerization or cyclic oligomer formation.^57,58^ This effect has been seen before in the process of lactide polymerization catalyzed by Lewis-acids and other organocatalysts, where also bifunctional mechanisms were followed.^59^ For ε-CL and l-LA ROP, the rate-determining step is primarily governed by carbonyl activation. In the case of l-LA ROP, the carbonyl activation was slower due to the steric interference of methyl group. Then, the formation of a hydrogen bond to the carbonyl of l-LA, resulted in a more stabilized transition state leading to a slower conversion rate disregarding the acidity or functionality of the organocatalyst.^60^

**Fig. 8:**
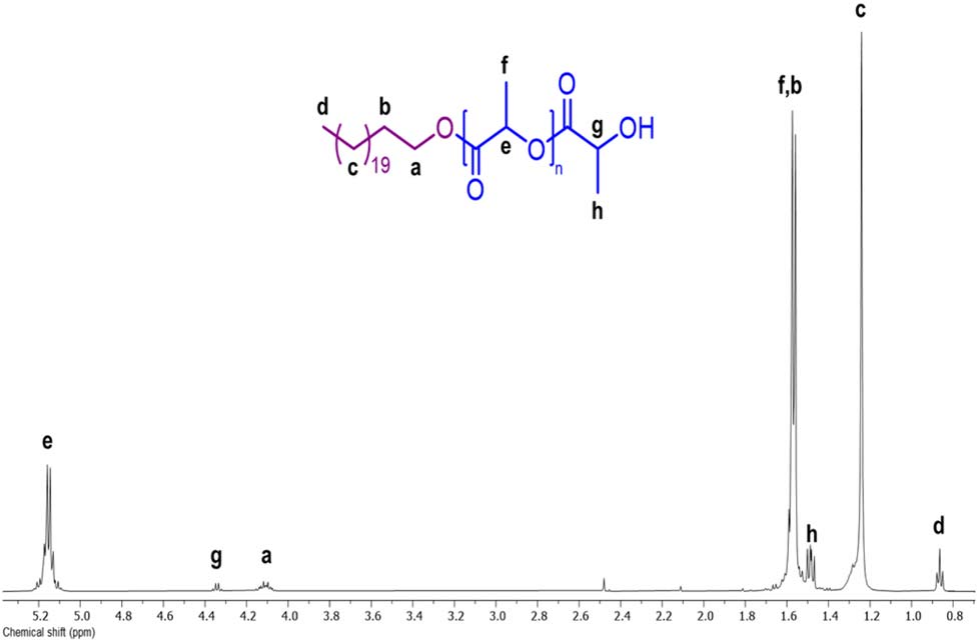
^1^H NMR spectrum at room temperature in CDCl_3_ of α-hydroxyl-ω-docosyl-PLLA obtained using citric acid as organocatalyst.

The ^1^H NMR result of α-hydroxyl-ω-docosyl-PLLA in Fig. 8 shows characteristic peaks of the methine and methylenes of the repeating unit of PLLA [–CH(CH_3_)–O–, 5.18 ppm (e), and –CH(CH_3_)–O–, 1.56 ppm (f), respectively]. There was also a signal for the PLLA hydroxyl terminal group [–CH(CH_3_)–OH, 4.33 ppm (g)]. The signals also correspond to methylenes of the docosyl main chain [–CH_2_–, 1.24 ppm (c)], methylene attached to the PLLA chain [–CH_2_–O–PLLA, 4.09 ppm (a)], and the terminal methyl [CH_3_–, 0.86 ppm (d)], and the incorporation of the 1-docosanol in the PLLA chain.

### Random PCL-*co*-PLLA copolymers

To obtain copolymers from CL and l-LA, reaction mixtures were prepared using the molar ratios established for homopolymerizations ([*M*]_0_/[*I*]_0_/[Cat]_0_ = 100/10/1). The initial monomer proportion was 10 mmol, which corresponded to the sum of the amounts of CL and l-LA. No significant variations were observed among the organocatalysts in the homopolymerization of l-LA, but citric acid was selected for its notable performance in the ROP of CL. Six copolymerization reactions were carried out using different molar ratios of CL and l-LA (Table 4). In all cases, the conversion of CL was greater than 98% after 24 hours.

**Table 4:** Copolymerization of ε-CL and l-LA using 7 citric acid as an organocatalyst^a^

Entry	Feed (% mol)		Conversion^b^ (%)		Composition^b^ (%)	
	CL	l-LA	CL	l-LA	CL	l-LA
1	95	5	99	94	97	3
2	90	10	99	94	95	5
3	80	20	99	94	89	11
4	70	30	99	92	82	18
5	60	40	99	92	72	28
6	50	50	99	91	63	37

a ROP in bulk. [*M*]_0_/[*I*]_0_/[Cat]_0_ = 100/10/1. [*M*]_0_ = 10 mmol. *T* = 150 °C *t* = 24 h.

b Determined by ^1^H NMR.

In the case of l-LA, the conversions never exceeded 95% even with higher proportions of l-LA. This disparity in conversion between the two monomers was evident in the final composition of the copolymer, which showed an imbalance of approximately 10% of each monomer with respect to the initial feed. For example, when the initial feed consisted of 80% CL and 20% l-LA, the final composition was 89% CL and 11% l-LA (Table 4, entry 3). This pattern was replicated for the rest of the reactions where the initial feed of l-LA was greater than 10% (Table 4, entries 3–6). For the two reactions in which the initial feed of l-LA was 5% and 10%, the final compositions of the monomers in the final polymer were 3% and 5%, respectively (Table 4, entries 1 and 2).

**Fig. 9:**
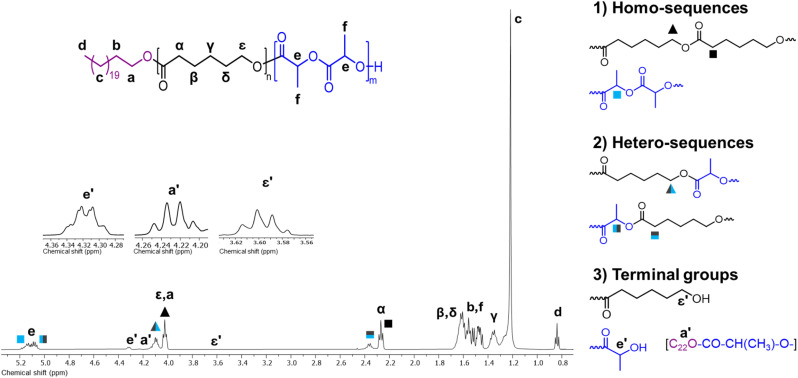
^1^H NMR spectrum (500 MHz) at room temperature in CDCl_3_ of PCL-*co*-PLLA (50–50%) (Table 3, entry 6).

The chemical composition of the copolymers was characterized by ^1^H and ^13^C NMR, which provided a comprehensive analysis of the molecular structures. Fig. 9 presents the ^1^H NMR spectrum for PCL-*co*-PLLA (50–50%) (Table 4, entry 6), which demonstrated the copolymerization of CL and l-LA through ROP. Three categories of sequences were identified in addition to the primary structure of the copolymer: (1) homo-sequences corresponded to signals indicating the attachment of (CL)–(CL) and (l-LA)–(l-LA). (2) Hetero-sequences as indicative of the union of CL unit to a l-LA unit, either the terminal hydroxyl of the CL attacked the carbonyl carbon of a l-LA molecule or *vice versa*. Consequently, the signals that were affected in the ^1^H NMR spectrum were the signals of the methylenes or methines adjacent to the carbonyl carbon or the oxygen at the propagation end. (3) Terminal groups exhibited chain ends. Examples include a low-intensity peak corresponding to the methylene of docosyl attached to an l-LA [ C_22_O–CO–CH(CH_3_)–O–, 4.22 ppm (a′)], signals of the terminal methylene of PCL [–CH_2_–OH, 3.60 ppm (ε′)], and a peak for the terminal methine of an l-LA unit [–CH(CH_3_)–O–, 4.31 ppm (e′)]. The latter signal was more intense, which indicated that a greater number of chains had α-hydroxyl terminal ends that corresponded to l-LA units.

The predominance of chains that had l-LA terminal ends was consistent with the reduced rate of l-LA conversion relative to CL. This phenomenon can be attributed to the premature consumption of CL, which resulted in l-LA as the final constituent to be incorporated into the polymer chain. The terminal end of l-LA was a secondary alcohol, and this structural feature influenced the propagation kinetics due to steric effects. Therefore, these propagation ends were consumed at a slower rate compared to the propagation ends of CL, which were primary alcohols.^61^

A powerful tool for determining each type of monomer sequence present in the copolymer is ^13^C NMR, particularly the signal region corresponding to the carbonyl carbons (165–175 ppm). A variety of potential sequences were identified, including one that demonstrated the occurrence of transesterification reactions, such as CLLLC (C = caproyl unit; L = lactidyl unit) at 169.75 ppm (Fig. S4). Finally, the average lengths of the lactide (LLL) and caprolactone (CL) units were evaluated only for PCL-*co*-PLLA (50–50%) (Table 4, entry 6), and respective values of 1.8 and 2.9 were obtained (see Experimental section). It has been established that for perfectly random copolymers, these values must be equal to 2. However, this was not the case, and the results indicated that a random block copolymer was obtained.

Fourier transform infrared spectroscopy (FT-IR) analysis was conducted on all prepared copolymers. Very slight changes were observed as the feed of l-LA increased in the reaction mixture, and the most evident changes were noted in the fingerprint region from 800 to 1200 cm^−1^ (Fig. S5). This region of the spectrum contained bands corresponding to the –C–O–C– and –C–(C=O)–O– groups present in both polymers. A slight broadening of the band was observed for the copolymers (Fig. S5b–d) with respect to the homopolymers (Fig. S5a and b). However, the band never became bimodal since the signals of both carbonyls overlapped at a similar wave number, even with equimolar feeds of both monomers.

**Fig. 10:**
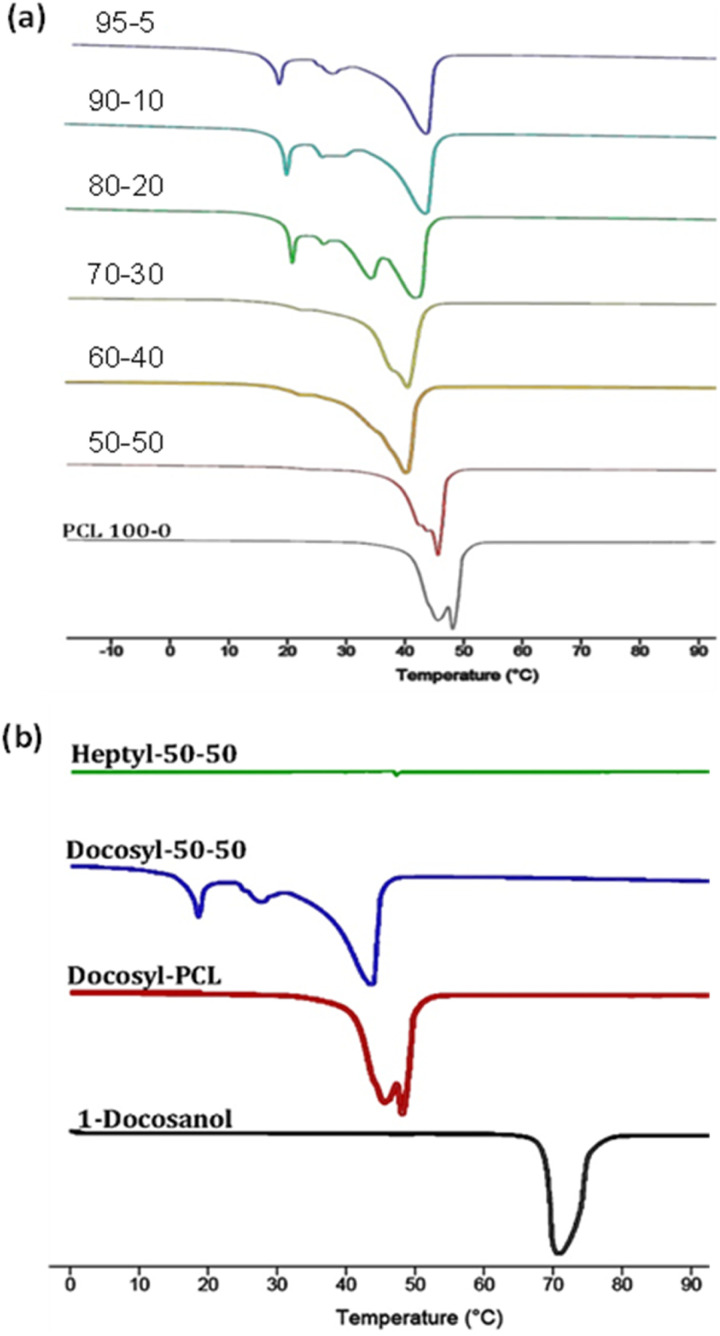
DSC thermograms of copolymers: (a) ω-C_22_-PCL-*co*-PLLA at different reaction mixtures, and (b) PCL-*co*-PLLA varying the terminal end (C_22_O– or C_7_O–).

The thermal properties of these copolymers were analyzed using DSC. In all cases, the melting temperatures (*T*_m_) varied slightly between 40 and 46 °C with no apparent pattern. However, a noticeable trend emerged as the percentage of l-LA in the final copolymer composition increased: the melting enthalpies (Δ*H*_m_) simultaneously decreased. Δ*H*_m_s decreased from 113 J g^−1^ for PCL to only 56 J g^−1^ for the copolymer containing 50% mol of l-LA (Table S2). l-LA has been observed to interfere with the crystalline microdomain in the PCL homopolymer (Fig. 10a).

Broadening of the *T*_m_ signal was observed as the percentage of l-LA increased in the copolymers, even with multimodal melting peaks. This suggested that higher percentages of l-LA contributed to the amorphous phase of the material or generated distortion in the crystalline microdomain of the PCL. Previous works on the synthesis of these copolymers reported the formation of fully amorphous materials, even with low feed percentages of l-LA (10 mol%).^49,62,63^ This phenomenon led to the hypothesis that the semi-crystalline behavior of the material in our samples was attributable to the initiator fraction (C_22_OH), which might be functioning as a nucleation agent or preserving its own crystalline microdomain (C_22_, aliphatic chain), despite being a component of a copolymer. This would be due to the large size of the aliphatic chain that it contains. Therefore, the multimodal behaviors of the thermograms may be attributable to the presence of distinct crystalline microdomains of the docosyl group fraction (C_22_), which may be surrounded by different sections of the copolymer that melt sequentially at different temperatures.

A series of additional experiments were conducted to substantiate this theory. In these experiments, an aliphatic alcohol with a smaller chain, 1-heptanol (C_7_OH), was used as the initiator in the copolymerization reaction. The reaction was carried out under the previously described conditions, and a copolymer with a CL : l-LA ratio of 57 : 43 was obtained after 24 hours. This copolymer was designated as ω-C_7_-PCL-*co*-PLLA, and DSC analysis indicated a completely amorphous material with no *T*_m_ present in the thermogram (Fig. 10b). These experiments validated the hypothesis that the semicrystalline behavior of the copolymers was attributable to the docosyl (C_22_) terminal fraction. Moreover, the results indicated that different crystalline microdomains can have different *T*_m_ based on their surrounding chemical environment.

**Fig. 11:**
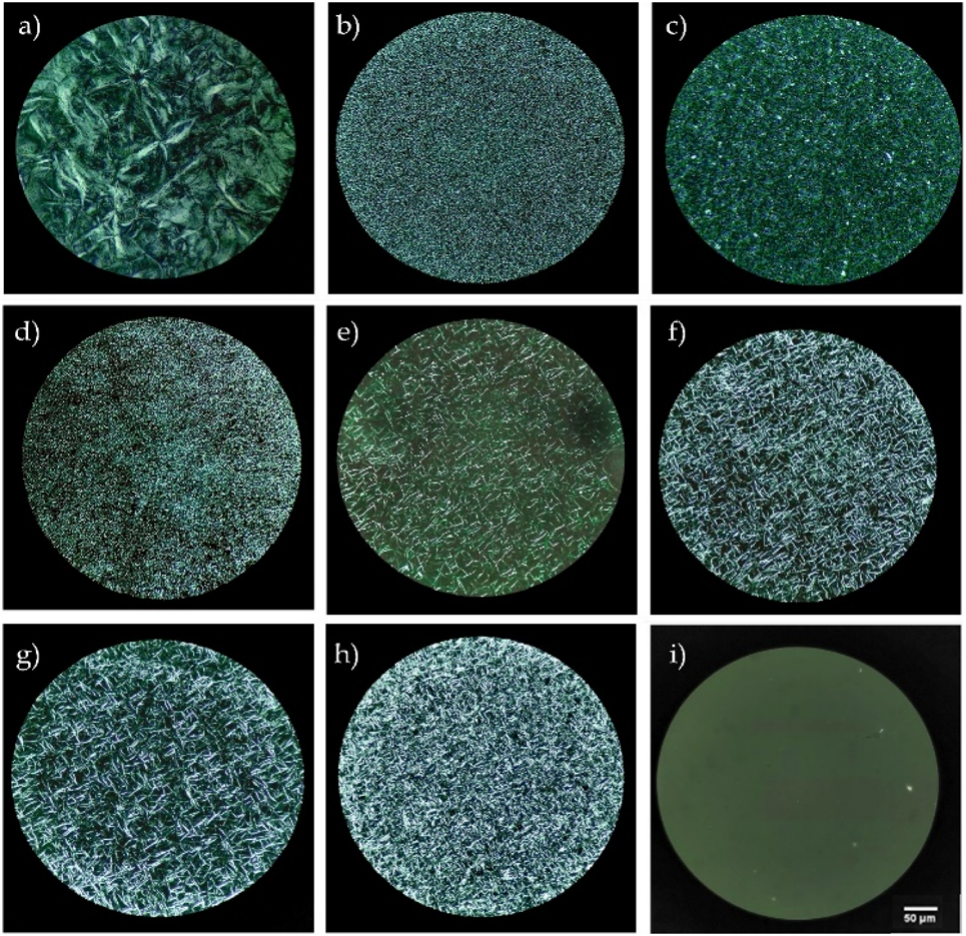
Polarized light microscopy (POM, 40× magnification) of (a) 1-docosanol (C_22_OH), (b) ω-C_22_-PCL, (c) ω-C_22_-PLLA, (d) ω-C_22_-PCL-*co*-PLLA 90–10, (e) ω-C_22_-PCL-*co*-PLLA 80–20, (f) ω-C_22_-PCL-*co*-PLLA 70–30, (g) ω-C_22_-PCL-*co*-PLLA 60–40, (h) ω-C_22_-PCL-*co*-PLLA 50–50, (i) ω-C_7_-PCL-*co*-PLLA 50–50.

The physical form of the 1-docosanol, homopolymers, and copolymers was visualized by polarized optical microscopy (POM), as shown in Fig. 11. Larger 1-docosanol spherulites were observed (Fig. 11a) in comparison with the PCL or PLLA homopolymers (Fig. 11b and c). The PCL-*co*-PLLA copolymers presented small spherulites that resembled those observed in homopolymers (Fig. 11d–h). In contrast, the POM of a copolymer with a heptyl group (C_7_) revealed no morphologies indicative of crystalline regions or amorphous material (Fig. 11i), which aligns with the thermal results with the absence of *T*_m_.

These observations reinforced the idea that the semi-crystalline behavior of the copolymers can be attributed to the docosyl (C_22_) terminal end. This finding was consistent with the report by Báez *et al.*^64^ on monodisperse oligomers of ω-C_22_-PCL, where the size of the monomer and dimer spherulites decreased compared to those observed in the C_22_OH sample. This observation suggests that the C_22_ content influences the size of the spherulites in the polymers. This implies that this terminal group functions as a nucleation agent that promotes semi-crystalline behavior of the copolymers obtained using C_22_OH as an initiator.

## Conclusions

A series of seven carboxylic acids were used as organocatalysts in the ROP of CL and l-LA and their copolymerization. The use of an aliphatic alcohol, 1-docosanol (C_22_OH), as an initiator has been demonstrated to accelerate the polymerization reaction rate and control the degree of polymerization (DP). The most efficient organocatalysts for the ROP of CL were those with p*k*_a_ values less than 4.

The reaction rates of the organocatalysts were proportional to the density of functional groups in their chemical structures, which was most evident for hydroxyl and carboxylic acid functionalities. Furthermore, the reaction rate was proportional to the amount of carboxylic acids present in the organocatalyst. The organocatalytic activity was highest for (COOH)_3_ [citric acid] (conversion = >98% time = 1 h 40 min, *T* = 150 °C), followed by (COOH)_2_ [succinic acid] (conversion = >98% time = 4 h, *T* = 150 °C) and COOH [butyric acid] (conversion = 45% time = 4 h, *T* = 150 °C). In the case of ROP of l-LA, the conversion was independent of the number of carboxylic acids present in the organocatalyst: (COOH)_3_ [citric acid] ≈ (COOH)_2_ [succinic acid] ≈ COOH [butyric acid].

PCL-*co*-PLLA random copolymers were synthesized using citric acid as the organocatalyst and C_22_OH as the initiator. The insertion of both monomers was determined, and the formation of various possible hetero-sequences was observed. Systematic increase in the l-LA fraction in the copolymerization reaction resulted in a decrease in the melting enthalpy (Δ*H*_m_) of the copolymer. However, despite high proportions of l-LA in the polymer structure, it never became amorphous. The semicrystalline behavior of the copolymers can be attributed to the C_22_O–(C=O)-PCL terminal group. The substitution of C_22_OH with 1-heptane (C_7_OH) as the initiator during the preparation of the 50–50 copolymer (PCL-*co*-PLLA) resulted in complete amorphous behavior of the copolymer C_7_O-PCL-*co*-PLLA according to the calorimetry and microscopy analyses.

## Author contributions

Juan Pablo Aldaba-Ramos: investigation, characterization, validation, formal analysis. Miriam Paola Barrera-Nava: characterization, writing-original draft. José Bonilla Cruz: characterization, financial support. Aurelio Ramírez Hernández: characterization, financial support. Alejandro Aparicio Saguilán: characterization, financial support. José E. Báez: conceptualization, supervision, characterization, writing-original draft, writing – review, funding acquisition.

## Conflicts of interest

There are no conflicts to declare.

## Supplementary Material

RA-015-D5RA07333B-s001

## Data Availability

The data supporting this article have been included as part of the suppplementary information (SI). Supplementary information is available. See DOI: https://doi.org/10.1039/d5ra07333b.
