# Supplementary material for: Homopolymerization and copolymerization of ε-caprolactone and l-lactide organocatalyzed by carboxylic acids with mono-, di-, and tri-functionality
Source: RSC Adv. 2025 Nov 6;15(50):43106–19. doi: 10.1039/d5ra07333b (PMC12590545; doi:10.1039/d5ra07333b)
Supplement: RA-015-D5RA07333B-s001 [file RA-015-D5RA07333B-s001.pdf]

Supplementary Information (SI) for RSC Advances.
This journal is © The Royal Society of Chemistry 2025

Supplementary Information

Homopolymerization and copolymerization of ε-caprolactone and L-lactide organocatalyzed by carboxylic acids with mono-, di-, and tri-functionality

Juan Pablo Aldaba-Ramos,[a] Miriam P. Barrera-Nava,[a] José Bonilla-Cruz,[b] Alejandro Aparicio-Saguilán,[c] Aurelio Ramírez-Hernández,[c] and José E. Báez *[a]

[a]Department of Chemistry, University of Guanajuato (UG), Noria Alta S/N, 36050 Guanajuato, Gto, Mexico.

[b]Advanced Functional Materials & Nanotechnology Group, Centro de Investigación en Materiales Avanzados S. C. (CIMAV-Unidad Monterrey), Av. Alianza Norte 202, Autopista Monterrey-Aeropuerto Km 10, PIIT, Apodaca-Nuevo León 66628 C.P., Mexico.

[c]Instituto de Química Aplicada, Universidad del Papaloapan, Circuito Central 200 Parque Industrial, San Juan Bautista Tuxtepec, C.P. 68301 Oaxaca, Mexico.

*Correspondence to: Prof. José E. Báez. Email: jebaez@ugto.mx

Table of contents

		Page
**Tables**		
	**Table S1.** Thermic properties of α-hydroxyl-ω-docosyl-PCL with different organocatalysts.	3
	**Table S2.** Thermic properties of PCL and copolymers PCL-*co*-PLLA using citric acid **7** as organocatalyst.	4
**Figures**		
	**Figure S1.** ^{13}C NMR spectrum (125 MHz) of α-hydroxyl-ω-docosyl-PCL (citric acid as organocatalyst, 100 min, 150 °C) in $CDCl_3$ at room temperature.	5
	**Figure S2.** Semi-logarithmic plot of the first-order behavior for the ROP reaction of CL, $Sn(Oct)_2$ (black line), and **7** (red line).	6
	**Figure S3.** Graphs of the dependence of M_n on the conversion percentage for the four most active organocatalysts: (a) citric acid **7**, (b) fumaric acid **5**, (c) malic acid **6**, and (d) succinic acid **4**.	7
	**Figure S4.** Expansion of the ^{13}C NMR spectrum (125 MHz) in the carbonyl carbon region of the PCL-*co*-PLLA copolymer (Table 3, Entry 6) and the triad assignments.	8
	**Figure S5.** FT-IR spectra of a) PCL, PCL-co-PLLA with CL/L-LA feed b) (90-10), c) (70-30), d) (50-50) and e) PLLA.	9
**Scheme**	**Scheme S1.** Chemical structure of PCL initiated by H_2O and detected by MALDI-TOF.	10

Table S1. Thermic properties of α-hydroxyl-ω-docosyl-PCL with different organocatalysts [a].

Organocatalyst	$M_{n(NMR)}$[b]	Conv.(%)[b]	T_m (°C)[c]	ΔH_m (J/g)[c]	ΔH_{mPCL} (J/g)[d]	x_i (%)[e]
**1**	650	45	45	116	57	42
**2**	1090	88	45	114	80	59
**3**	1080	85	45	112	78	57
**4**	1380	>98	46	111	83	61
**5**	1230	>98	46	114	84	62
**6**	1260	>98	48	114	85	63
**7**	1320	>98	47	113	85	63

[a]ROP of ε-CL with 1-docosanol as initiator at 150°C, ratio $[M]_0/[I]_0/[Cat]_0$ = 100 : 10 : 1 (mmolar ratio= 10 : 1 : 0.1).
[b]Obtained by ^{1}H NMR analysis.
[c]Obtained by DSC analysis, second heating.
[d]Obtained by the equation ΔH_{mPC}L=(ΔH_m)•(weight fraction of PCL).
[e]Using the value of 135.3 J/g for a PCL 100% crystalline, the crystallinity of PCL (x_{PCL}) was calculated.

Table S2. Thermic properties of PCL and copolymers PCL-*co*-PLLA using citric acid **7** as organocatalyst [a].

Entry	Composition (%)		T_m (°C)[b]	ΔH_m (J/g)[b]	ΔH_{mPCL} (J/g)[c]	x_i (%)[d]
	CL	L-LA				
**1**	100	0	46	113	113	83
**2**	97	3	46	97	94	69
**3**	95	5	40	85	80	59
**4**	89	11	40	83	73	54
**5**	82	18	42	70	57	42
**6**	72	28	43	62	44	32
**7**	63	37	44	56	35	25

[a]ROP in bulk. $[M]_0/[I]_0/[Cat]_0$= 100/10/1. $[M]_0$= 10 mmol, T= 150 °C, t= 24 h.
[b]Obtained by DSC analysis, second heating.
[c]Obtained by the equation ΔH_{mPC}L=(ΔH_m)•(weight fraction of PCL).
[d]Using the value of 135.3 J/g for a PCL 100% crystalline, the crystallinity of PCL (x_{PCL}) was calculated.

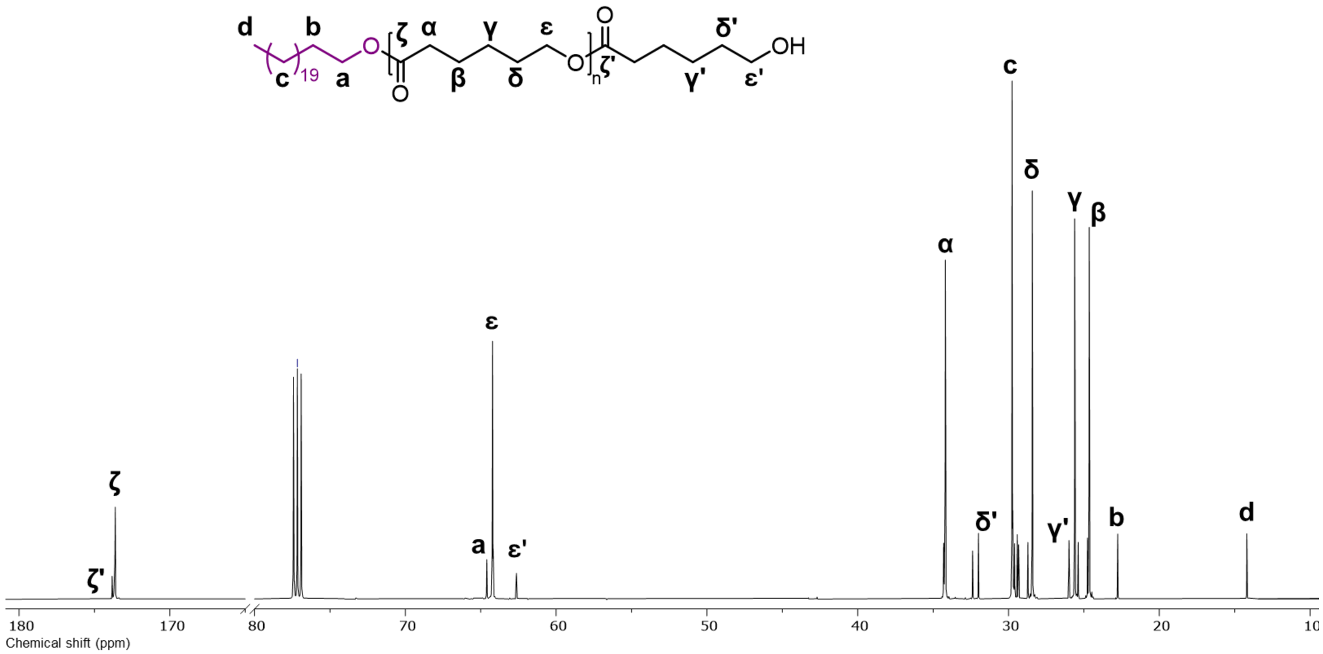

Figure S1. ^{13}C NMR spectrum (125 MHz) of α-hydroxyl-ω-docosyl-PCL (citric acid as organocatalyst, 100 min, 150 °C) in $CDCl_3$ at room temperature.

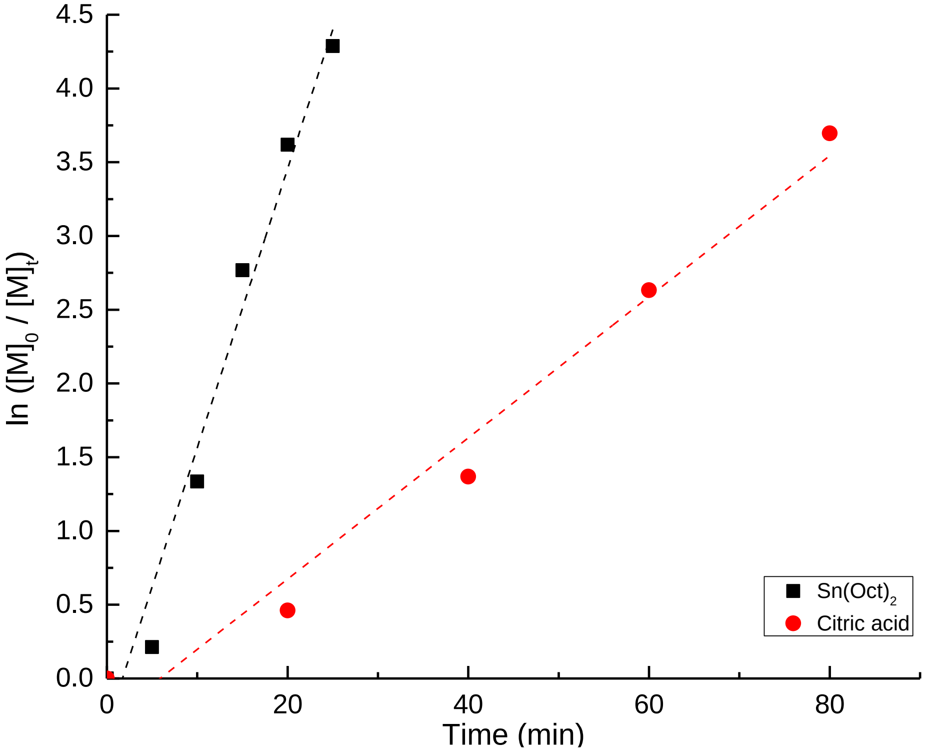

Figure S2. Semi-logarithmic plot of the first-order behavior for the ROP reaction of CL, $Sn(Oct)_2$ (black line), and **7** (red line).

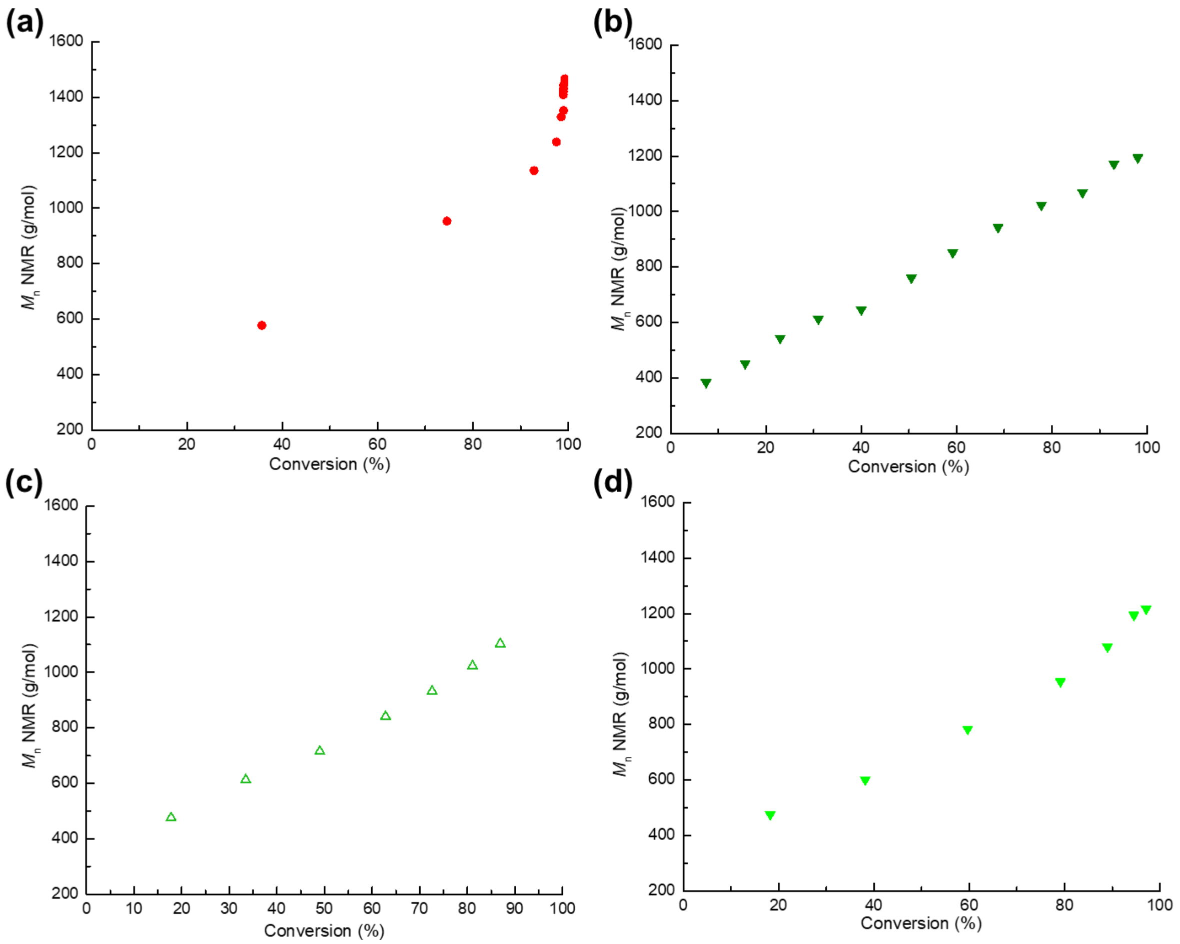

Figure S3. Graphs of the dependence of M_n on the conversion percentage for the four most active organocatalysts: (a) citric acid **7**, (b) fumaric acid **5**, (c) malic acid **6**, and (d) succinic acid **4**.

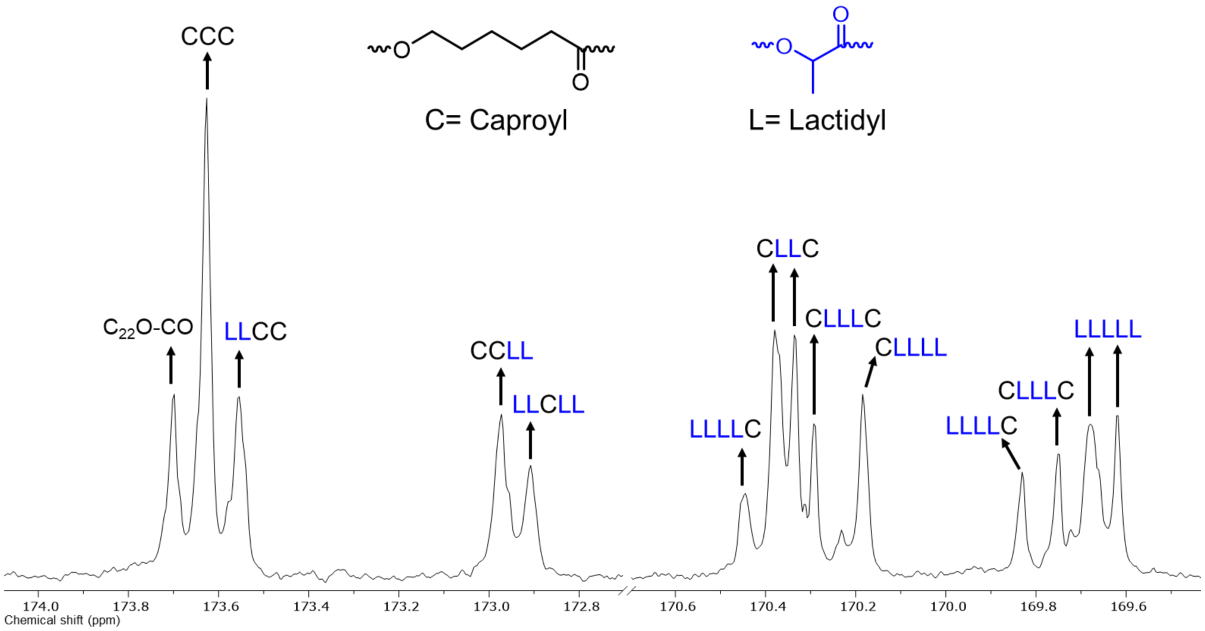

Figure S4. Expansion of the ^{13}C NMR spectrum (125 MHz) in the carbonyl carbon region of the PCL-*co*-PLLA copolymer (Table 3, Entry 6) and the triad assignments (acquisition time=18 h).

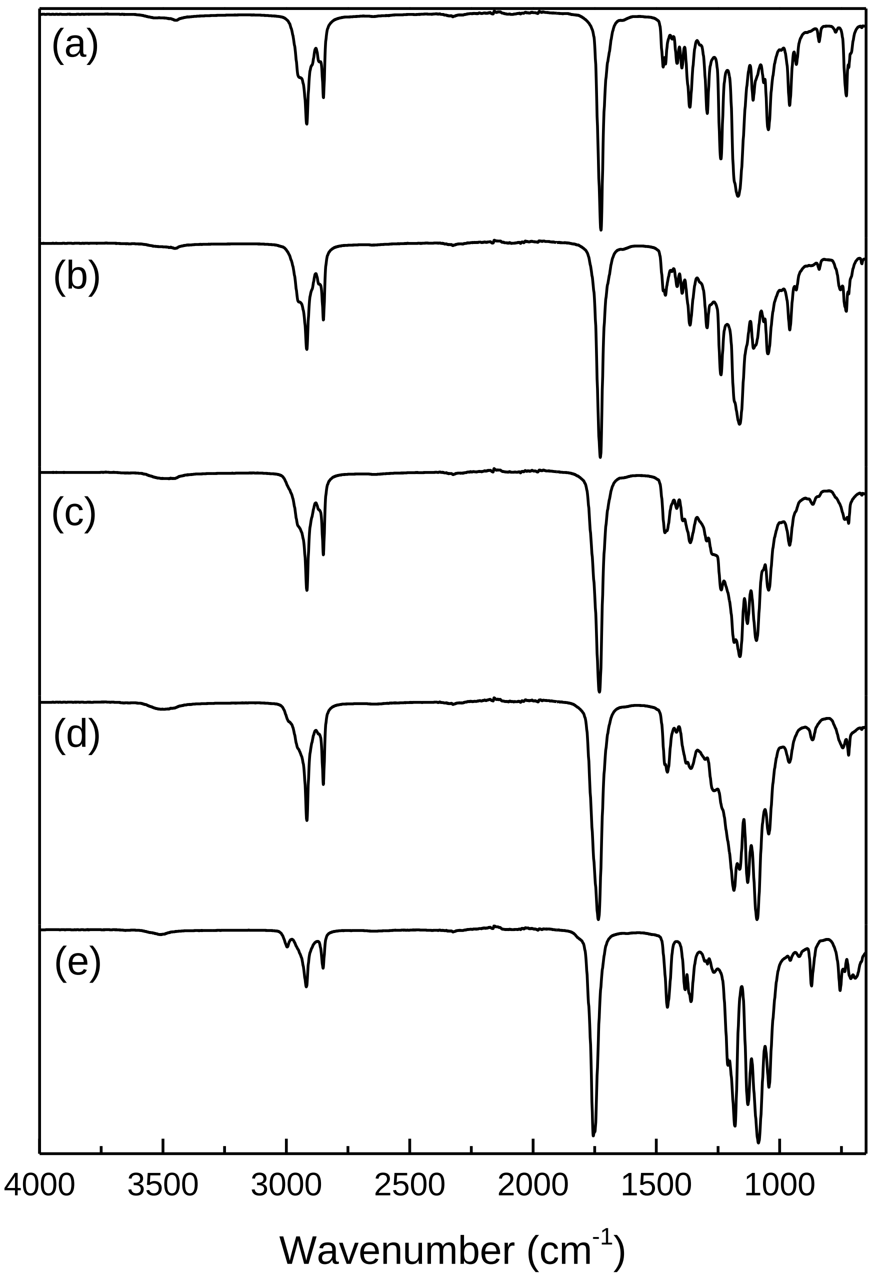

Figure S5. FT-IR spectra of a) PCL, PCL-co-PLLA with CL/L-LA feed b) (90-10), c) (70-30), d) (50-50) and e) PLLA.

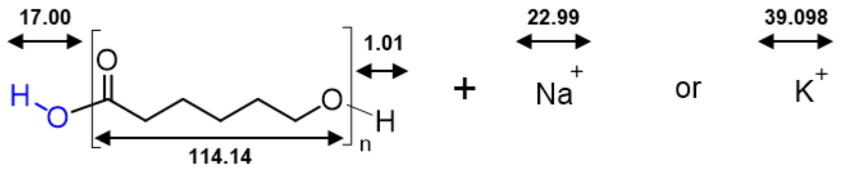

Scheme S1. Chemical structure of PCL initiated by H_2O and detected by MALDI-TOF.

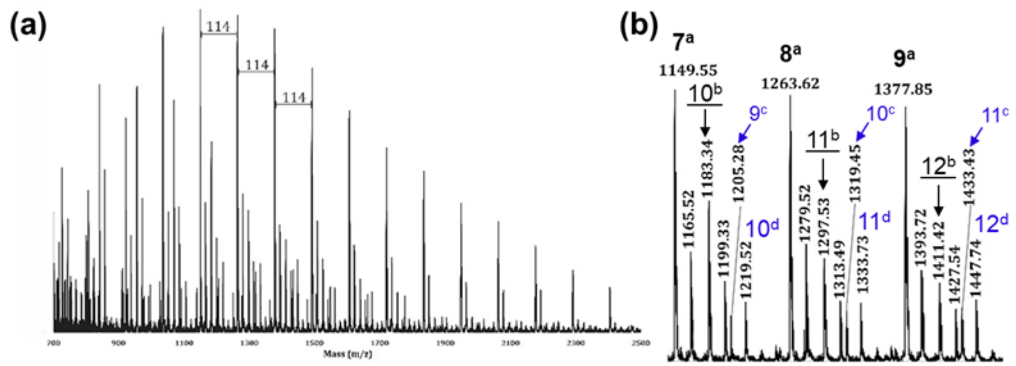

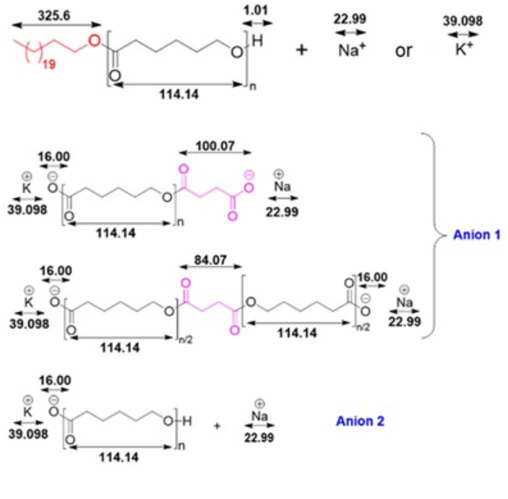

Theoretical values

	Cation	n			
		7[a]	8[a]	9[a]	10[a]
PCL initiated by $C_{22}OH$	Na^+	1148.57	1262.71	1376.85	1490.99
	K^+	1164.68	1278.82	1392.96	1507.10
	Cation	10[b]	11[b]	12[b]	13[b]
PCL initiated by H_2O	Na^+	1182.40	1296.54	1410.68	1524.82
	K^+	1198.41	1312.55	1426.69	1540.83
		9[c]	10[c]	11[c]	12[c]
**Anion 1**	-	1205.42	1319.56	1433.70	1547.84
		10[d]	11[d]	12[d]	13[d]
**Anion 2**	-	1220.49	1334.56	1448.63	1562.70
